# Mechanochemistry-Accessed MOF Polymorphism Revealed by an Integrated 3DED/DFT-Assisted Rietveld Analysis

**DOI:** 10.1021/acs.inorgchem.6c02037

**Published:** 2026-08-06

**Authors:** Giorgio Cagossi, Andrea Daolio, Dario Giovanardi, Iryna Andrusenko, Enrico Mugnaioli, Lucia Carlucci, Paolo P. Mazzeo, Alessia Bacchi, Paolo Pelagatti

**Affiliations:** † Department of Chemical Sciences, Life Science and Environmental Sustainability, 9370 University of Parma, Parco Area delle Scienze 17/A, Parma 43124, Italy; ‡ Centre for Instrumentation Sharing of the University of Pisa (CISUP), 9310 University of Pisa, Lungarno Pacinotti 43/44, Pisa 56426, Italy; § Department of Earth Sciences, University of Pisa, Via S. Maria 53, Pisa 56126, Italy; ∥ Department of Chemistry, Università Degli Studi di Milano, Via Golgi 19, Milano 20133, Italy; ⊥ Biopharmanet-TEC, University of Parma, via Parco Area Delle Scienze 27/A, Parma 43124, Italy; # Interuniversity Consortium of Chemical Reactivity and Catalysis (C.I.R.C.C.), Via Ulpiani 27, Bari 70126, Italy

## Abstract

A combined 3DED, plane-wave DFT, and Rietveld refinement workflow uncovers the first example of interlayer-twisting polymorphism in a pillared MOF. While the parent material, **PUM198**, a Zn-based mixed-ligand MOF, was previously synthesized under solvothermal conditions, mechanochemistry selectively affords a new polymorph, **PUM198-m**. Although the two phases share identical composition and connectivity, they differ in symmetry, layer arrangement, pore architecture, and topology. Accessible only as a microcrystalline powder, **PUM198-m** is unsuitable for conventional single-crystal X-ray diffraction, and its flexibility and solvent-filled pores preclude structure solution from the XRPD data. The crystal structure was therefore derived from a multitechnique blueprint in which the starting model was derived from 3DED data and subsequently refined through iterative DFT-assisted Rietveld analysis to describe the as-synthesized, guest-loaded material. This integrated workflow provides a broadly applicable structural elucidation strategy for flexible MOFs that are mechanochemically synthesized, for which conventional crystallographic analysis fails.

## Graphical abstract

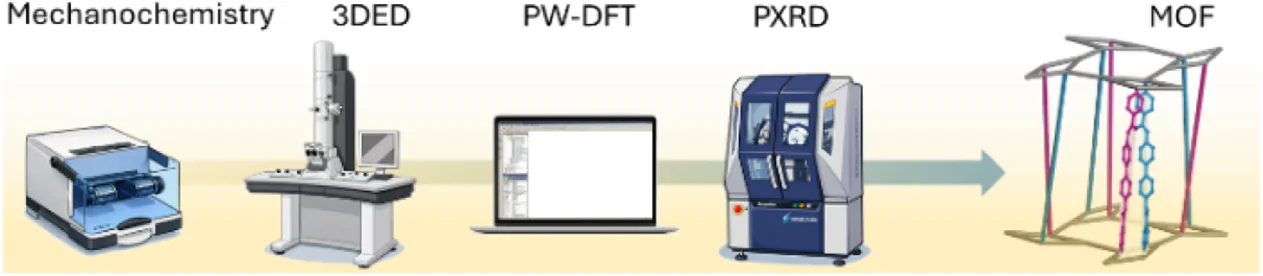

## Introduction

Mechanochemistry has emerged as a powerful synthetic strategy in which chemical transformations are driven by mechanical energy, typically through the grinding of solid reactants.^1^ By minimizing or eliminating solvent use, this approach reduces the environmental impact, improves reaction efficiency, and lowers production costs. Beyond its sustainability advantages, mechanochemistry can also unlock reaction pathways that are inaccessible or inefficient under conventional solution-based conditions.^1–4^

Metal–organic frameworks (MOFs) represent a prominent class of porous crystalline materials composed of metal-containing secondary building units (SBUs) interconnected by organic linkers.^5–7^ Their structural diversity and tunable porosity underpin applications in gas storage, separation, catalysis, and sensing.^8–13^ The transformative impact of MOF chemistry was recently recognized by the 2025 Nobel Prize in Chemistry, awarded to Kitagawa, Yaghi, and Robson for their pioneering contributions to the field.^14^ A wide range of archetypal MOFs, primarily synthesized via wet chemistry, have now been successfully reproduced by mechanochemical protocols, including copper­(II) isonicotinate,^15^ HKUST-1,^16^ MOF-5,^17^ UiO-66,^18^ and several zeolitic imidazolate frameworks (ZIFs),^19–21^ with no compromise in terms of crystallinity with respect to their solvothermal counterparts.

Among MOFs, those with a pillared-layered architecture (PL-MOFs) occupy a particularly versatile niche. These materials consist of two-dimensional metal-carboxylate layers interconnected by N-containing axial pillars,^22^ like bis-pyridines, bis-amines, bis-imidazoles, or pyrazines, thus combining two chemically distinct linker types within a single framework. This modular design enables extensive functionalization and structural tunability.^23–26^ The successful mechanochemical preparation of PL-MOFs such as [Zn_2_(5-aip)_2_(bipy)][Bibr ref27] (5-aip = 5-aminoisophthalate, bipy = 4,4́′-bipyridine), reflects the growing recognition that mechanochemical strategies can be even applied to increasingly complex framework architectures. Moreover, we have recently achieved the dimensionality growth of 1D-coordination polymers containing dpe (dpe = 1,2-bis­(4-pyridyl)­ethylene) to 3D PL-MOFs via insertion of terephthalate under mechanochemical conditions.^28^ Previously, our group developed, under solvothermal conditions, a new family of PL-MOFs named PUM (Parma University Materials). These MOFs contain a series of extended bis-amidic pillars,^29^ in which amide groups serve as hydrogen-bond donors and acceptors, creating internal recognition sites for supramolecular interactions. Such features enhance affinity toward a wide range of guest molecules, including natural phenolic terpenoids^30–32^ and nitrogen-containing heterocycles,^33^ leading to controlled guest release. Among the PUM family,^34^ **PUM198**, made of terephthalate spacers and rigid bis-pyridine-bis-amide pillars (**L1**, Figure 1), was revealed to be ideal for adsorbing polyaromatic heterocycles through noncovalent interactions.^35^ Based on the potential applications offered by PUM materials, the development of a mechanochemical synthetic route is highly desirable to overcome the typical limitations of solvothermal synthesis. Herein, we explored the mechanochemical synthesis of the previously reported solvothermal synthesis of **PUM198**, consistently and selectively yielding a different polymorphic form, **PUM198-m** (Figure 1). When prepared via mechanochemistry, the material is produced as a microcrystalline powder, precluding structure determination by conventional single-crystal X-ray diffraction (SCXRD). Three-dimensional electron diffraction (3DED) data collection performed in TEM on individual submicrocrystals directly harvested from the milling jar allows for complete structural elucidation, overcoming the intrinsic limitations associated with nonrecrystallizable materials. Efficiency and accuracy of the structure solution and refinement are increased when data are collected in association with beam precession^36^ and/or with continuous sample rotation (cRED).^37,38^ Recently, the possibility of applying serial electron diffraction (SerialED) on MOFs has also been explored.^39^ The combination of mechanochemical synthesis and electron crystallography therefore represents a broadly applicable and transformative strategy for the routine structural characterization of nano- and microcrystalline framework materials.^40–42^

**Figure 1:**
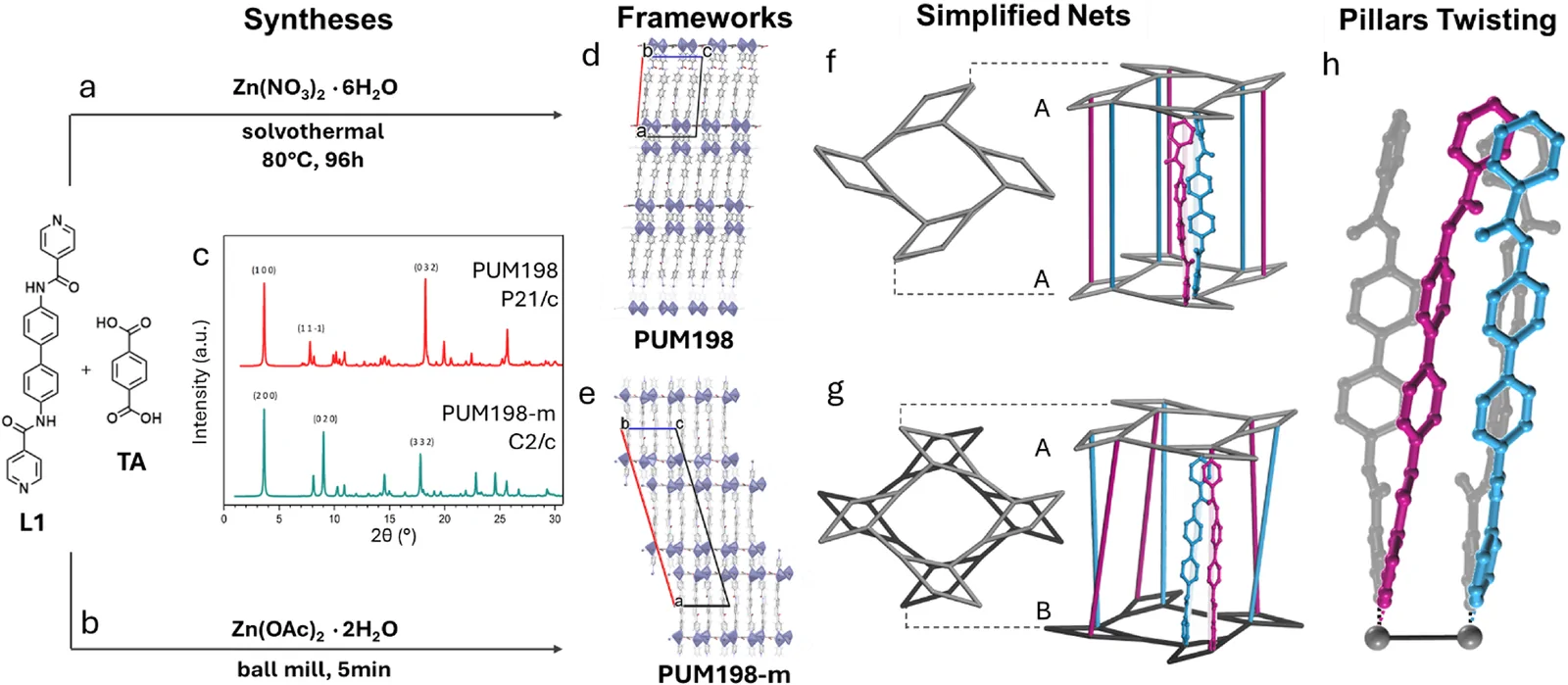
(a) Synthesis details for **PUM198** (solvothermal) and (b) **PUM198-m** (ball-milling) with a scheme of the ligands used. (c) Calculated PXRD patterns of **PUM198** (red line) and **PUM198-m** (green line) with represented plane families highlighted. (d) Framework visualizations of **PUM198** (Refcode: ZILDEG^30^) and (e) **PUM198-m** with Zn atoms depicted in polyhedral style and nonmetal atoms depicted as sticks. H atoms and guest molecules are removed for the sake of clarity. Double interpenetration is highlighted by representing the second framework in transparency. Unit cells are reported to emphasize the *P*-centered (**PUM198**) against the *C*-centered (**PUM198-m**) structure. (f) Simplified nets of **PUM198** and (g) **PUM198-m** highlighting the rotation of the 2D-squared layers containing **TA** ^2 –^ anions with consequent pillar twisting. (h) Pillars twisting in **PUM198-m** highlighted by superimposition of two adjacent **L1** ligands (colored) with the same ligands in **PUM198** (gray transparency). Zn atoms, reported as gray balls, are superimposed to stress the pillar orientation differences.

Polymorphism in MOFs is well-documented,^43–48^ yet cases where distinct synthetic routes produce polymorphs through subtle framework-level conformational reorganization remain uncommon.^49^ In most cases, structural variations arise from linker flexibility,^50^ changes in SBU geometry or connectivity,^51,52^ or kinetic trapping of alternative node assemblies.^53^ Although linker design has been shown to guide framework connectivity,^54^ polymorphism originating from different spatial orientations of the same rigid linker under distinct synthetic protocols is a rare condition.

PL-MOFs, such as **PUM198**, are known to exhibit pronounced structural flexibility, often undergoing substantial lattice distortions upon guest inclusion or removal.^30,31,33^ Such breathing behavior can involve changes in interlayer spacing, pore geometry, and even symmetry or unit-cell metrics. Host–guest interactions are typically governed by weak, noncovalent forces, so the incorporated solvent molecules can be readily evacuated under mild vacuum conditions, frequently inducing framework rearrangements.^55^ Such responsiveness complicates structural characterization, as 3DED data collected in a high vacuum environment may not directly reflect the as-synthesized, solvent-loaded state of the material.

Here, we introduce a robust and transferable multitechnique workflow that integrates electron diffraction, Plane Wave-Density Functional Theory (PW-DFT) calculations, and Rietveld refinement into a unified structure-solution strategy that may serve as a blueprint for the structural elucidation of mechanochemically derived microcrystalline frameworks. The framework model obtained from 3DED served as the starting platform for iterative DFT-assisted Rietveld refinement, allowing the correct structural incorporation of dimethylformamide (DMF) guest molecules into the crystallographic model. The guest content was quantified by Nuclear Magnetic Resonance (NMR) spectroscopy and by thermogravimetric analysis (TGA), ultimately yielding a fully resolved and experimentally validated crystal structure of **PUM198-m** and granting access to its “as-synthesized” framework.

## Experimental and Computational Section

### Materials and Methods

All reagents and solvents were of high purity and used as received, unless otherwise indicated. *N*,*N*-Dimethylformamide (99.9% purity) was purchased from Carlo Erba. Ligand **L1** was synthesized according to a previously described procedure.^29^

### Synthesis

All mechanochemical syntheses were performed in a Retsch MM400 mixer mill, utilizing 5 mL stainless steel jars and balls of the same material (Ø = 10 mm, *w* ≈ 4 g). Liquid-Assisted Grinding (LAG) conditions were applied, using a small volume of DMF. In the following, the symbol η refers to the ratio between the volume of DMF (μL) and the mass of solids (mg). Zn­(OAc)_2_·2H_2_O (55 mg, 0.25 mmol), **L1** (98 mg, 0.25 mmol), terephthalic acid (42 mg, 0.25 mmol), and DMF (200 μL, η = 0.66 μL/mg) were introduced into the jar along with one ball. The jar was shaken for 5 min at 30 Hz. The product was retrieved, washed three times with DMF (1 mL), and dried in air to obtain a yellow powder. Yield: 91% (based on Zn content).

### Powder X-ray Diffraction

PXRD data for phase checking and quantification were collected in the Bragg–Brentano geometry with Cu Kα radiation on a Rigaku SmartLab XE diffractometer equipped with a solid-state HyPix-3000 2D detector. The samples were placed on a glass sample holder and measured with a scan rate of 5° min^–1^ in the range 1.5° ≤ 2θ ≤ 50°. Every sample was vertically aligned with respect to the incoming beam to moderate the vertical sample displacement. A length-limiting slit of 10 mm was used as a compromise to enhance X-ray flux over the sample, while 2.5° Soller slits improved the peak profile and reduced reflection overlap. Data collection for Rietveld Refinement was performed in Debye–Scherrer geometry with the sample loaded in a sealed borosilicate glass capillary (Ø 0.3 mm, 80 mm length) mounted on the capillary spinner and measured under continuous rotation (2 Hz) to minimize preferred orientation effects. PXRD data analyses were performed with TOPAS V6.^56^ Pawley refinement was performed considering a seventh-order Chebyshev polynomial function to fit the background and a TCHZ function for the definition of the line profile (Figures S6–S7). The same input layout was also used for the Rietveld Refinement (Figures S8–S9) with the structure obtained from DFT optimization included as a rigid body with rotation and translation free to vary within a limited constrained range (±10°) in the first refinement. Once refined, rotation and translation of the rigid body were kept fixed, while intramolecular rotations of the ligands were refined, although constrained within a limited range (±10°). After the framework optimization, DMF positions were refined by the constrained H-bond lengths and angles with a tolerance of 10%. All atoms were isotropically refined.

### TEM and Three-Dimensional Electron Diffraction

A small tip of powdered sample (less than 1 mg) was gently ground between two glass plates and then sprinkled on a carbon-coated Cu TEM grid. The sample was analyzed with a JEOL JEM F200 Multipurpose TEM, working at 200 kV and equipped with a Schottky field emission gun (FEG). The pressure inside the column was about 2 × 10^–5^ Pa, a high vacuum guaranteed by a dedicated superionic pump with a capacity of 150 L/s. 3DED was performed in scanning transmission electron microscopy (STEM) mode after defocusing the beam in order to achieve pseudoparallel illumination on the sample.^57,58^ 3DED data were collected with a beam size of about 30 nm in diameter, obtained using a 10 μm C2 condenser aperture and the highest probe size (10). Data collections were performed in stepwise mode (tilt step = 1°), and the covered tilt ranged up to 100°. After each tilt, a diffraction pattern was acquired, and the crystal position was tracked by defocused STEM imaging, performed with high-angle annular dark-field (HAADF-STEM). The camera length was set to 300 mm, allowing for a resolution in real space of about 1.0 Å. The beam was precessed around the optical axis by an angle of 1° to properly integrate every reflection over the excitation error.^59^ Precession was obtained using a Nanomegas Digistar P1000 device. 3DED data were recorded by an ASI CheeTah hybrid-pixel electron detector (512 × 512 pixels) and analyzed with the program PETS2.0.^60^ The structure solution was performed in two steps. Inorganic clusters were solved ab initio by standard direct methods (SDM), while position and conformation of organic linker molecules were determined by simulated annealing (SA). Both methods are implemented in the SIR2014 software package.^61^ The molecules of terephthalate and bis-pyridine–bis-amide were extracted from the solvothermal **PUM198** structure (Refcode: ZILDEG). This method has been previously applied for the determination of covalent organic frameworks,^62^ hybrid perovskites,^63^ and important pharmaceuticals.^64,65^ The achieved structural data were refined with a fully kinematical approximation, i.e., neglecting dynamical scattering and assuming that *I*_hkl_ is proportional to |*F*_hkl_|^2^.

### PW-DFT Calculation Details

Starting from the 3DED-determined atomic positions with the addition of 5 DMF molecules (manually added with the Materials Studio program^66^ to mimic the position of guest molecules in the parent solvothermal **PUM198** structure), the structure was optimized by means of the *PW*-DFT program CASTEP 2023.1.^67^ The choice of parameters employed in the geometry optimization was tailored to provide a general scheme applicable to the DFT-Assisted Rietveld refinement of MOFs while optimizing for computational resources and is in line with other works on the subject.^68–70^ The wave function was expanded in plane waves to a kinetic energy cutoff of 400 eV. The electronic structure was sampled at the Γ point. The exchange-correlation functional of Perdew–Burke–Ernzerhof (PBE)^71^ was used with the dispersion correction of Tkatchenko–Scheffler (TS).^72^ The core–valence interaction was modeled with a norm-conserving pseudopotential generated on-the-fly as implemented in CASTEP. Convergence was accepted when residual atomic forces reached <1 × 10^–2^ eV Å^–1^, with SCF convergence accepted at <1 × 10^–6^ eV. The Pulay density mixing scheme was employed as the electronic minimizer algorithm. The DFT optimizations had the unit cell restrained to the PXRD-indexed cell parameters, while all the atomic positions could be optimized.

### Thermogravimetric Analysis

TGA analyses were performed with a PerkinElmer TGA 8000 instrument (mass sample: 1–3 mg) by means of a Pt crucible under an air flux (30 mL/min) in the temperature range of 30–500 °C at 10 °C/min. Higher temperatures were not applied to avoid possible crucible damage due to the high metal content of the samples.

### ^1^H NMR Spectroscopy

^1^H NMR spectra were recorded on a Bruker Avance spectrometer operating at 400 MHz at 25 °C after dissolving the sample in CF_3_COOD (TFA-d) and diluting it with (CD_3_)_2_SO (DMSO-*d*_6_). Chemical shifts (δ) are expressed in ppm relative to the residual peak of deuterated DMSO (^1^H = 2.50 ppm).

Semiquantitative ^1^H NMR analyses were performed to estimate the relative amount of coordinated and/or included DMF with respect to the MOF. After complete dissolution of the sample, the integrals were referenced to well-resolved, nonoverlapping proton signals of the two organic ligands, which were considered together as one asymmetric unit. The characteristic singlet of DMF (N–CH_3_) at ca. δ = 7.95 ppm was integrated and compared with pyridinic C–H proton resonances of the ligands at ca. δ = 9.1 ppm. The integrals were normalized according to the number of protons contributing to each signal, allowing determination of the molar ratio between DMF and the asymmetric unit.

### Topological Analysis

The topological analysis of **PUM198-m** and **PUM198** was performed using ToposPro software, applying the standard simplification procedure.^73^ The search for structures exhibiting the same topological type and degree of interpenetration as **PUM198-m** was carried out by accessing the TopCryst database through the website resource (https://topcryst.com/).^74^

### BET Measurements

Adsorption measurements were carried out using a BELSORP MAX X porosimeter employing N_2_ 5.0 gas. BET was estimated using BELmaster software in the *P*/*P*_0_ range of 0.01–0.1 bar.

### Crystallographic Details

## Results and Discussion

### Synthesis

**PUM198** is a PL-MOF made of Zn­(II) nodes coordinated by terephthalate (**TA** ^2–^) linkers and the bis-pyridine–bis-amide pillar **L1.** The resulting framework is built around a double-pillared bimetallic SBU of formula [Zn_2_(μ-COO)_2_(κ^1^-COO)_2_(py)_4_], where the two carboxylate groups of each **TA** ^2–^ anion behave differently. These units arrange together into two-dimensional layers, subsequently pillared by the axial bis-amidic ligands, generating the three-dimensional framework (Figure 1a).

The original solvothermal synthesis employed zinc nitrate hexahydrate as the metal source (Figure 1a). Direct transfer of these conditions to mechanochemical synthesis using DMF as a LAG additive (30 Hz, one 10 mm Ø SS ball) was unsuccessful. Addition of a base (a stoichiometric amount of triethylamine corresponding to the complete neutralization of **TA**) after 5 min of milling resulted in the formation of multiple phases but not the desired **PUM198-m**. Replacing zinc nitrate with the more basic zinc acetate dihydrate proved crucial for successful mechanochemical synthesis (Figure 1b). Under solvothermal conditions, DMF can partially decompose into basic species such as dimethylamine, which promotes the slow deprotonation of the dicarboxylic linker and enables MOF formation from Zn­(NO_3_)_2_·6H_2_O. In contrast, under mechanochemical conditions, this source of in situ basicity is absent, making the more basic Zn­(OAc)_2_·2H_2_O ideal to facilitate linker deprotonation and framework assembly.^75^ The optimized procedure involved loading zinc acetate together with both ligands in a 1:1:1 stoichiometric ratio (Zn^2+^:**TA**:**L1**) and addition of a small volume of DMF. Remarkably, complete conversion to **PUM198-m** as the sole crystalline phase was achieved within 5 min, affording a bright yellow dry powder with high yield (91% after washing and air drying). Milling times beyond 5 min did not improve the yield, although the material demonstrated good mechanical stability and crystallinity, even after prolonged milling time (several hours, see Supporting Information and Figures S11–S12). The LAG ratio of η = 0.66 μL/mg proved optimal, whereas values lower than 0.50 were unsuccessful, leading to mixtures of phases. Importantly, strict adherence to the 1:1:1 stoichiometric ratio was essential to obtain **PUM198-m** as a single phase, as deviation from this ratio led to the formation of secondary undefined crystalline phases. This stoichiometric requirement reflects the 1:1:1 metal-to-ligand composition of the **PUM198-m** framework.

### **PUM198-m** Structural Determination

#### 3D ED-TEM

Independent 3DED data sets were collected from several crystalline fragments smaller than 1 μm (Figure 3b). All data sets exhibited the systematic extinction condition *h*0*l*: *l* = 2*n*, consistent with a *C*-centered monoclinic lattice. The data set covering the largest tilt range and free of artifacts arising from polycrystallinity was selected for structure determination. The refined unit-cell parameters were approximately *a* = 48.3(2), *b* = 19.7(2), *c* = 15.2(4) Å, with β = 97.4(3)°. Structure solution was achieved in the centrosymmetric space group *C*2/*c*.

The mechanochemically obtained framework retains the same connectivity as the solvothermal analogue **PUM198** (Figure 1d–g). A small variation involves the SBU that now has the formula [Zn_2_(μ-COO)_2_(κ^1^-COO)­(κ^2^-COO)­(**L1**)_4_], where the two nonbridging carboxylates behave differently, one being κ^1^-monodentate and the other being κ^2^-chelating (Figure S1). However, differences in linker orientation induce a displacement and rotation of adjacent layers in **PUM198-m** (Figure 1f–g), resulting in a symmetry change from *P*2_1_/*c* (**PUM198**) to *C*2/*c* (Table 1), with a consequent doubling of the cell volume and *a*-axis. In **PUM198**, the bis-amidic pillars are parallel and generate a regular A–A–A stacking sequence of the orthogonal zinc-carboxylate 2D layers. In contrast, in **PUM198-m**, the two pillars adopt a twisted arrangement, coiling around one another by approximately 90°, thereby connecting orthogonal squared layers rotated by 90° relative to each other, producing an A–B–A–B stacking pattern consistent with the *C*-centered symmetry (Figure 1). Like its parent phase, **PUM198-m** exhibits 2-fold interpenetration of class IIa (i.e., the two frameworks are related by a center of inversion) (Figure S4), with both nets being binodal 3,5-connected. The differences lie in the topology of the 3,5-c nets, particularly in the point symbols of the 3-c nodes, which are (4.6^2^) in **PUM198** and (4.6.8) in **PUM198-m**, corresponding to the **fet** and **fsc**-3,5-*Ibam* topologies, respectively (Figure S15).^76^ A search of the TopCryst database^74^ for 2-fold interpenetrated structures with the **fsc**-3,5-*Ibam* topology returned only three PL-MOF structures (refcodes: BOWSIQ, EWESAC, and QOYZUD), whose topological analysis revealed a twisting of the metal–carboxylate layers similar to that observed in **PUM198-m**.^77^

**Table 1:** Crystallographic Details for **PUM198-m** as Refined by Rietveld Refinement

	PUM198-m
CCDC Deposition Number	2541762
Phase Percentage	100%
Chemical Formula	C_64_H_44_N_8_O_12_Zn_2_·5(C_3_H_7_NO)
Formula Weight	1552.6616
*Z*	8
Temperature (K)	298(2)
Space Group	*C*2/*c*
*a* (Å)	50.9649(2)
*b* (Å)	19.6008(3)
*c* (Å)	15.5652(3)
β (°)	107.4035(6)
*V* (Å^3^)	14837.1(4)
ρ (g/cm^*3*^)	1.444
Rbragg (%)	4.04
Rp (%)	6.78
Rwp (%)	8.76
Refinement Method	Rietveld
Measurement Device Type	Rigaku Smartlab XE
Radiation Wavelength (Å)	1.5406
Measurement Method	Continuous Scan

The framework defines two distinct rectangular channels running along the *c*-axis (Figure 2a). The larger channels display window dimensions of approximately 18.3 × 9.8 Å^2^, whereas the smaller one measures about 6.6 × 9.8 Å^2^ (Figure 2b). The calculated void volume (32%) closely matches that of **PUM198** (31%), indicating that interlayer twisting preserves the overall porosity. The experimentally measured BET surface areas of the activated materials indicate an increase for **PUM198-m**, from 46 to 75 m^2^/g (see Supporting Information and Figures S16), although the absolute values remain rather low for both materials. Nevertheless, the reorientation of the linkers alters the amide exposure within the channels (Figure 1h).

**Figure 2:**
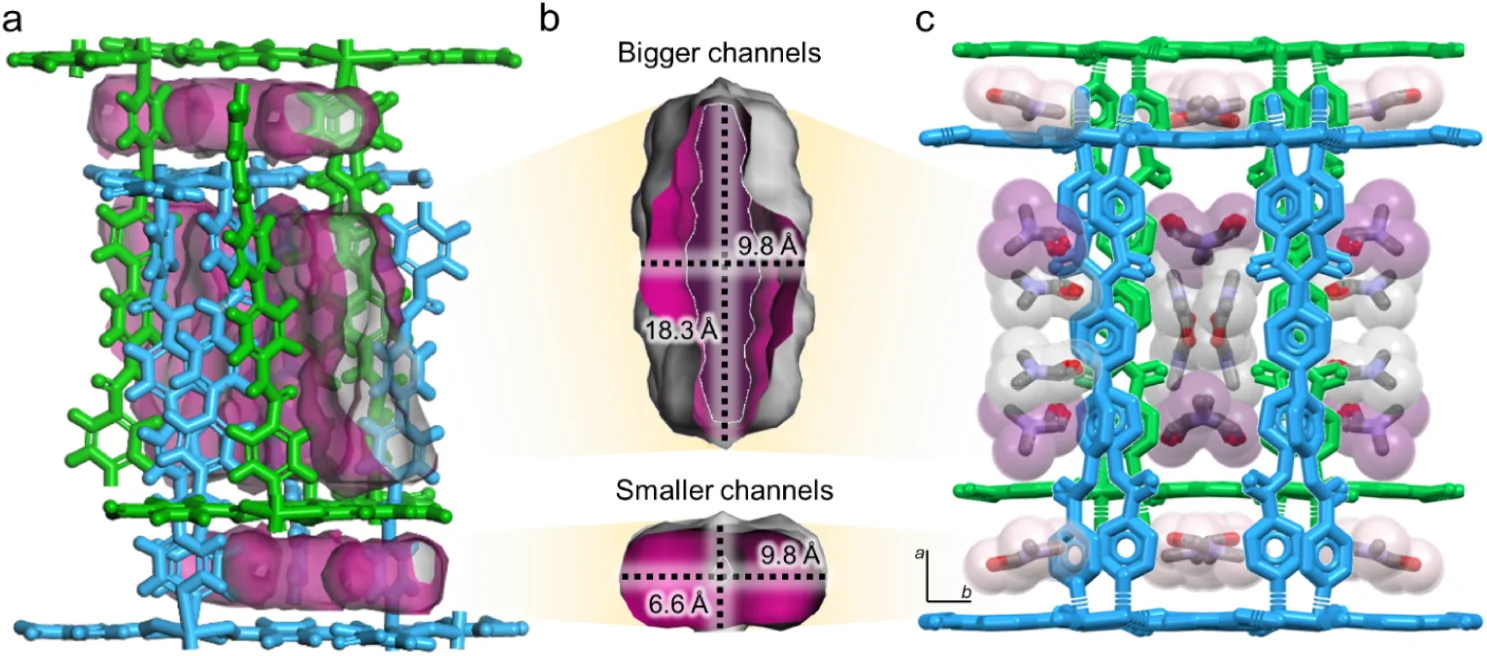
(a) Porosity of **PUM198-m**. Interpenetrated nets are highlighted with different colors. Channel surfaces are highlighted in purple. (b) Channel voids along the *c*-axis with superimposed pore dimensions. (c) Detail of the DMF molecules hosted within the framework along the *c*-axis: symmetry-related molecules are haloed with the same color.

Although structural modifications arising from subtle twisting and/or sliding motions in interpenetrated frames have been reported,^78^ polymorphism arising from plane twisting in PL-MOFs is unprecedented and markedly different from the more commonly reported structural variants originating from pillar flexibility.^79^

While the 3DED-derived model unambiguously establishes the framework connectivity and topology, it does not resolve the positions of the native DMF guest molecules, which may have an impact on the whole framework arrangement. Their presence within the pores of the mechanochemically synthesized product was confirmed by ^1^H NMR spectroscopy (Figure S13) and TGA analysis (Figure S14). The absence of electron density corresponding to DMF likely results from the high-vacuum conditions inherent to electron diffraction, which promote solvent evacuation during data acquisition and induce the associated framework distortion.

### DFT-Assisted Rietveld Refinement

PXRD data were collected on freshly mechanochemically synthesized **PUM198-m,** loaded in a 0.3 mm Ø glass capillary with high-resolution Soller slits to improve angular resolution. Initial indexing using the unit cell parameters derived by 3D ED-TEM returned only a modest agreement with the experimental pattern (Rwp 23.85), symptomatic of substantial framework distortion upon solvent removal from the pores due to the electron diffraction setup. The set of unit cell parameters was validated by Pawley refinement (*C*2/*c*; *a* 50.98(2), *b* 19.60(3), *c* 15.57(2) Å, β 107.42(2)°), with a significantly improved match against the experimental data (Rwp 5.19) (Figures S2, S6–S7).

The structural model with the atomic positions determined from 3DED was used as a starting platform for the DFT optimization (CASTEP^67^) with the inclusion of five DMF molecules *per* asymmetric unit, as determined by ^1^H NMR and TGA. The amount of guest molecules added is compatible with the volume of DMF that was inserted into the jar. The DMF molecules were initially positioned into the **PUM198-m** pores, mimicking those found in the parent **PUM198**. During the DFT optimization, the unit-cell parameters were fixed to the PXRD-indexed values, while all atomic positions were allowed to relax (see the Experimental and Computational Section for details).

Rietveld refinement was finally performed against the PXRD data using the DFT-optimized structure. The framework was treated as a rigid body in TOPAS^56^ with fixed bond lengths and angles, while limited conformational flexibility (≤10°) was allowed to account for internal ligand relaxation and rotation about coordination axes. Coordination bond lengths were further refined to account for thermal effects. Finally, the positions of the DMF molecules were optimized, allowing for the refinement of the hydrogen bond distances and angles, as evidenced in Figure 2c (see also Figure S5).

After DFT optimization and Rietveld refinement (Figures S8−S9, the final structural model of **PUM198-m** was found to closely match the original as-synthesized **PUM198-m** structure. The high degree of agreement between the experimental PXRD pattern and those simulated from the different structural models further supports this conclusion (as shown in Figures S3, S10, S17, and S18).

A schematic workflow followed for the structural elucidation of **PUM198-m** is shown in Figure 3.

**Figure 3:**
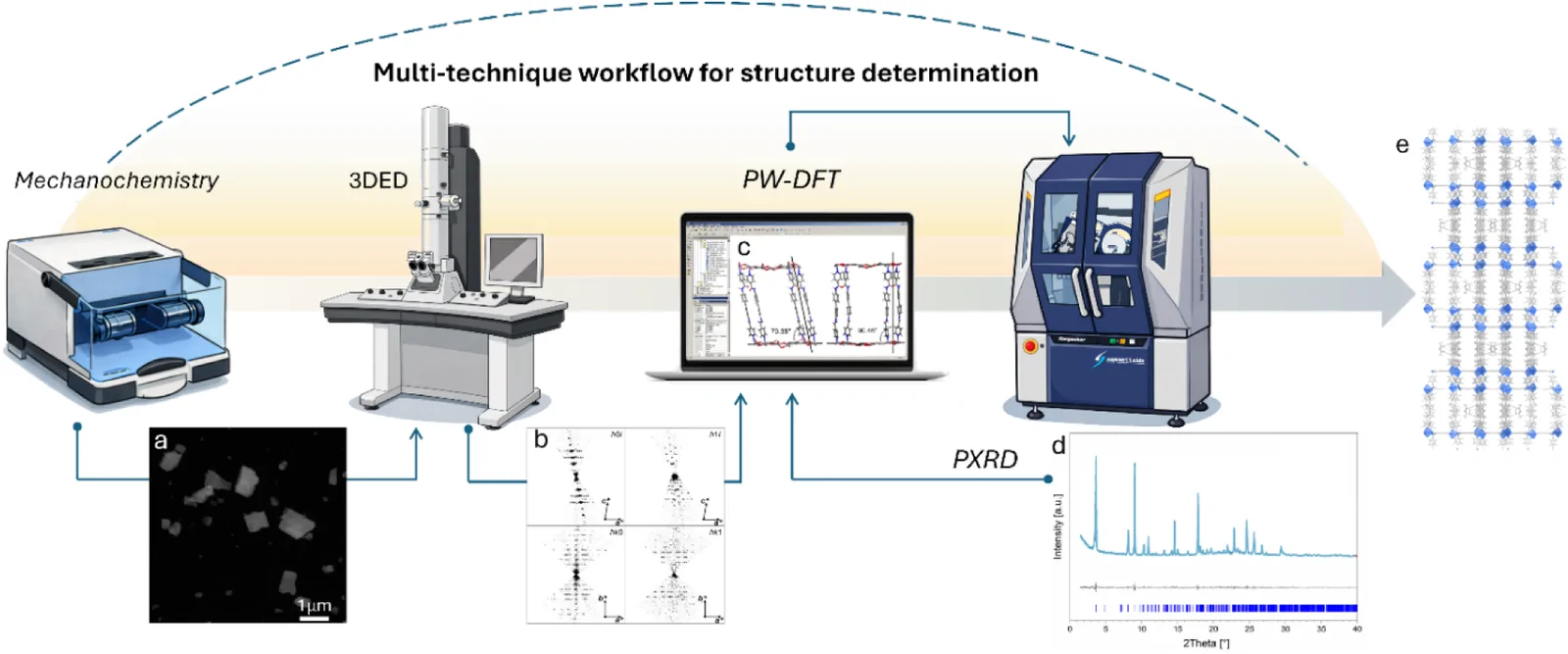
Schematic workflow followed for the structural elucidation of **PUM198-m**. The sample was directly obtained from mechanochemistry as microcrystalline powder (a), which was directly measured by 3D ED-TEM (b). The cell parameters were first refined against PXRD experimental data collected on the freshly synthesized sample, then the initial model structure with the addition of DMF molecules was minimized by the PW-DFT algorithm (c) and finally Rietveld refined against the PXRD experimental pattern (d), thus leading to the final model of **PUM198-m** (e).

## Conclusions

The integration of three-dimensional electron diffraction with DFT-assisted Rietveld refinement constitutes a robust and broadly applicable strategy for the structural elucidation of mechanochemically derived nano- and microcrystalline frameworks. By combining reciprocal-space information from 3DED with computationally guided refinement of powder diffraction data, this workflow overcomes the intrinsic limitations of single-crystal diffraction and enables reliable modeling of flexible, guest-containing materials. Beyond resolving the structure of **PUM198-m**, this multitechnique approach establishes a transferable blueprint for characterizing complex framework materials that cannot be recrystallized. Importantly, its application here reveals a previously unknown polymorph arising from a subtle reorientation of rigid pillars, which induces interlayer twisting and symmetry modulation without altering the overall connectivity or composition, a type of structural variation not reported for PL-MOFs. This finding highlights how mechanochemistry can selectively access alternative structural states encoded within a given framework, uncovering latent degrees of freedom in MOF architectures and expanding the structural landscape accessible to porous materials.

## Supplementary Material



## Notes

Experimental, crystallographic, X-ray traces, and computational details, mechanical stability, ^1^H NMR spectrum, TGA trace of**PUM198-m**, and BET traces of **PUM198** and **PUM198-m** (PDF)

## ∇

Andrea Daolio, Department of Chemistry, Stockholm University, Svante Arrhenius väg 16C, Stockholm, Sweden, SE-106 91
