# Supplementary material for: Mechanochemistry-Accessed MOF Polymorphism Revealed by an Integrated 3DED/DFT-Assisted Rietveld Analysis
Source: Inorg Chem. 2026 Aug 6;65(32):18639–47. doi: 10.1021/acs.inorgchem.6c02037 (PMC13488400; doi:10.1021/acs.inorgchem.6c02037)
Supplement: Supplementary file 1 [file ic6c02037_si_001.pdf]

# Supporting Information

## Mechanochemistry-Accessed MOF Polymorphism Revealed by an Integrated 3DED/DFT-Assisted Rietveld Analysis

*Giorgio Cagossi,[a] Andrea Daolio,[a] Dario Giovanardi,[a] Iryna Andrusenko,[b] Enrico Mugnaioli,[b,c] Lucia Carlucci,[d] Paolo P. Mazzeo,* [a,e] Alessia Bacchi,[a,e] Paolo Pelagatti* [a,f]*

[a] G. Cagossi, Dr. A. Daolio, Dr. D. Giovanardi, Prof. P. P. Mazzeo, Prof. A. Bacchi, Prof. P. Pelagatti
Department of Chemical Sciences, Life Science and Environmental Sustainability
University of Parma
Parco Area delle Scienze 17/A, 43124, Parma, Italy
E-mail: paolo.pelagatti@unipr.it

[b] Dr. I. Andrusenko
Centre for Instrumentation Sharing of the University of Pisa (CISUP)
University of Pisa
Lungarno Pacinotti 43/44, 56426 Pisa, Italy

[c] Prof. E. Mugnaioli
Department of Earth Sciences
University of Pisa
Via S. Maria 53, 56126 Pisa, Italy

[d] Prof. L. Carlucci
Department of Chemistry,
Università degli Studi di Milano,
Via Golgi 19, 20133 Milano, Italy

[e] Prof. A. Bacchi, Prof. P. P. Mazzeo
Biopharmanet-TEC,
University of Parma
Via Parco Area delle Scienze 27/A, 43124 Parma, Italy

[f] Prof. P. Pelagatti
Interuniversity Consortium of Chemical reactivity and Catalysis (C.I.R.C.C.)
Via Ulpiani 27, 70126 Bari, Italy

General....3
Materials and methods....3
Powder X-Ray Diffraction....3
TEM and Three-Dimensional Electron Diffraction....3
PW-DFT Calculation details....4
Thermogravimetric analysis....4
$^{1}$H-NMR....4
Topological analysis....4
$N_2$ Adsorption Isotherms....4
Synthesis....4
Synthesis of Ligand L1....4
Mechanochemical synthesis of PUM198-m....5
Experimental....5
Crystallographic details....5
Structural analysis of PUM198-m....9
Polymorphs stability testing....10
Powder X-Ray Diffraction Analyses....10
Thermogravimetric analysis....14
Topological details....15
$N_2$ Adsorption Isotherms....15
Structural comparison between resolution phases....16
References....16

# General

## Materials and methods

All reagents and solvents were of high purity and used as received if not otherwise indicated. N,N-dimethylformamide (99.9% purity) was purchased from Carlo Erba.

## Powder X-Ray Diffraction

PXRD data for phase checking and quantification were collected in the Bragg–Brentano geometry with Cu Kα radiation on a Rigaku SmartLab XE diffractometer equipped with a solid-state HyPix-3000 2D detector. The samples were placed on a glass sample holder and measured with a scan rate of 5° $min^{-1}$ in the range 1.5° ≤ 2θ ≤ 50°). Every sample was vertically aligned with respect to the incoming beam to moderate the vertical sample displacement. A length-limiting slit of 10 mm was used as a compromise to enhance X-ray flux over the sample, while 2.5° Soller slits improved the peak profile and reduced reflection overlap. Data collection for Rietveld Refinement was performed in Debye–Scherrer geometry with the sample loaded in a sealed borosilicate glass capillary (Ø 0.3 mm, 80 mm length) mounted on the capillary spinner and measured under continuous rotation (2 Hz) to minimize preferred orientation effects. PXRD data analyses were performed with TOPAS V6.[1] Pawley refinement was performed considering a seventh-order Chebyshev polynomial function to fit the background and a TCHZ function for the definition of the line profile.
The same input layout was also used for the Rietveld Refinement with the structure obtained from DFT optimization included as rigid body with rotation and translation free to vary within a limited constrained range (± 10°) in the first refinement. Once refined, rotation and translation of the rigid body was kept fixed while intramolecular rotations of the ligands were refined although constrained in a limited range (± 10°). After the frameworks optimization, DMF positions were refined by the constrained H-bond lengths and angles with a tolerance of 10%. All atoms were isotropically refined.

## TEM and Three-Dimensional Electron Diffraction

A small tip of powdered sample (less than 1 mg) was gently ground between two glass plates and then sprinkled on a carbon coated Cu TEM grid. The sample was analysed with a JEOL JEM F200 Multipurpose TEM, working at 200 kV and equipped with a Schottky field emission gun (FEG). The pressure inside the column was about $2 \cdot 10^{-5}$ Pa, a high vacuum guaranteed by a dedicated super ionic pump with capacity of 150 L/s. 3DED was performed in scanning transmission electron microscopy (STEM) mode after defocusing the beam in order to have a pseudo-parallel illumination on the sample[2,3]. 3DED data were collected with a beam size of about 30 nm in diameter, obtained using a 10 μm C2 condenser aperture and the highest probe size (10). Data collections were performed in stepwise mode (tilt step = 1°) and the covered tilt ranges up to 100°. After each tilt, a diffraction pattern was acquired, and the crystal position was tracked by defocused STEM imaging, performed with high-angle annular dark-field (HAADF-STEM). Camera length was set to 300 mm, allowing for a resolution in real space of about 1.0 Å. The beam was precessed around the optical axis by an angle of 1° to proper integrate every reflection over the excitation error[4].Precession was obtained using a Nanomegas Digistar P1000 device. 3DED data were recorded by an ASI CheTaah hybrid-pixel electron detector (512 x 512 pixels) and analysed with the program PETS2.0[5]. The structure solution was performed in two steps. Inorganic clusters were solved ab-initio by standard direct methods (SDM), while position and conformation of organic linker molecules were determined by simulated annealing (SA). Both methods are implemented in the SIR2014 software package[6]. The molecules of terephthalate and bis-pyridine-bis-amide were extracted from solvothermal PUM198 structure (Refcode: ZILDEG). This method has been previuosly applied for the determination of a covalent organic framework,[7] hybrid perovskite[8] and important pharmaceuticals.[9,10] The achieved structural data were refined with a fully kinematical approximation, i.e., neglecting dynamical scattering and assuming that $I_{hkl}$ is proportional to $|F_{hkl}|^2$.

## PW-DFT Calculation details

Starting from the 3DED determined atomic positions with the addition of 5 DMF molecules (manually added with the Material Studio program[11]) the structure was optimized by means of the *PW*-DFT program CASTEP[12]. The wave function was expanded in plane waves to a kinetic energy cutoff of 400 eV. The electronic structure was sampled at the Γ point. The exchange-correlation functional of Perdew–Burke–Ernzerhof (PBE)[13] was used with the dispersion correction of Tkatchenko–Scheffler (TS)[14]. The core-valence interaction was modelled with a norm-conversing pseudopotential generated on-the-fly as implemented in CASTEP. Convergence was accepted when residual atomic forces reached <1 × $10^{-2}$ eV $Å^{-1}$, with SCF convergence accepted at <1 × $10^{-6}$ eV. The DFT optimizations had the unit-cell restrained to the PXRD indexed cell parameters while all the molecules could be optimized.

## Thermogravimetric analysis

TGA analyses were performed with a PerkinElmer TGA 8000 instrument (mass sample: 1–3 mg) by the means of a Pt crucible under air flux (30 mL/min) in the temperature range of 30–500 °C at 10 °C/min. Higher temperatures were not applied to avoid possible crucible damage due to the high metal content of the samples.

## $^1$H-NMR

$^1$H-NMR spectra were recorded on a Bruker Avance spectrometer operating for 1H at 400 MHz at 25 °C after dissolving the sample in CF3COOD (TFA-d) and diluting it with (CD3)2SO (DMSO-d6). Chemical shifts (δ) are expressed in ppm relative to the residual peak of deuterated DMSO (1H = 2.50 ppm)

Semi-quantitative $^1$H-NMR analyses were performed to estimate the relative amount of coordinated and/or included DMF with respect to the MOF. After complete dissolution of the sample, the integrals were referenced to well-resolved, non-overlapping proton signals of the two organic ligands, which were considered together as one asymmetric unit. The characteristic singlet of DMF (N–$CH_3$) at ca. δ = 7.95 ppm was integrated and compared with pyridinic C-H proton resonances of the ligands at ca. δ = 9.1 ppm. The integrals were normalized according to the number of protons contributing to each signal, allowing determination of the molar ratio between DMF and the asymmetric unit. The resulting values represent an average composition and should be regarded as semi-quantitative due to possible differences in relaxation times and baseline uncertainties.

## Topological analysis

The topological analysis of **PUM198-m** and **PUM198** was performed using the ToposPro software applying the standard simplification procedure.[15] The search for structures of the same topological type and interpenetration degree of **PUM198-m** was performed accessing the TopCryst database through the website resource (https://topcryst.com/).[16]

## $N_2$ Adsorption Isotherms

Bulk of **PUM198** and **PUM198-m** were activated washing the samples with acetone and letting the powder/crystals soaking for 24h. The obtained material was then transferred into two adsorption cells and treated under vacuum at 120°C for 3 days by the means of BELPrep-VAC-III.

# Synthesis

## Synthesis of Ligand L1

Ligand **L1** was synthesized according to the procedure previously described.[17]

## Mechanochemical synthesis of PUM198-m

All mechanochemical synthesis were performed in a Retsch MM400 ball mill, utilizing 5 mL stainless steel jars and balls of the same material (ø = 10mm, w ≈ 4g).

$Zn(OAc)_2·2H_2O$ (55mg, 0.5mmol), **L1** (98mg, 0.25mmol) and terephtalic acid (42mg, 0.25mmol) and DMF (200 µL) were introduced into the jar along with one ball. The jar was shaken for 5 min at 30Hz. The product was retrieved, washed three times with DMF (1 mL) and dried in air to obtain a yellow powder. Yield: 91% (based on Zn content). Milling times beyond 5 minutes did not improve the yield, although the material demonstrated excellent mechanical stability and crystallinity, even after prolonged milling time (several hours). The LAG ratio of $\eta$ = 0.66 µL/mg proved optimal, whereas values lower than 0.50 revealed unsuccessful, leading to mixtures of phases. Importantly, strict adherence to the 1:1:1 stoichiometric ratio was essential to obtain **PUM198-m** as a single phase, as deviation from this ratio led to the formation of secondary undefined crystalline phases. This stoichiometric requirement reflects the 1:1:1 metal-to-ligand composition of the **PUM198-m** framework.

# Experimental

### Crystallographic details

**Table S1**. Crystallographic details for **PUM198-m** as refined by Rietveld Refinement.

| | ***PUM198-m*** |
|---|---|
| *CCDC Deposition Number* | 2541762 |
| *Phase Percentage* | 100 % |
| *Chemical Formula* | $C_{64}H_{44}N_8O_{12}Zn_2•5(C_3H_7NO)$ |
| *Formula Weight* | 1552.6616 |
| *Z* | 8 |
| *Temperature (K)* | 298(2) |
| *Space Group* | C2/c |
| *a (Å)* | 50.9649(2) |
| *b (Å)* | 19.6008(3) |
| *c (Å)* | 15.5652(3) |
| *β (°)* | 107.4035(6) |
| *V (Å³)* | 14837.1(4) |
| *ρ (g/cm³)* | 1.444 |
| *Rbragg (%)* | 4.04 |
| *Rp (%)* | 6.78 |
| *Rwp (%)* | 8.76 |
| *Refinement Method* | Rietveld |
| *Measurement Device Type* | Rigaku Smartlab XE |
| *Radiation Wavelength (Å)* | 1.5406 |
| *Measurement Method* | Continous Scan |

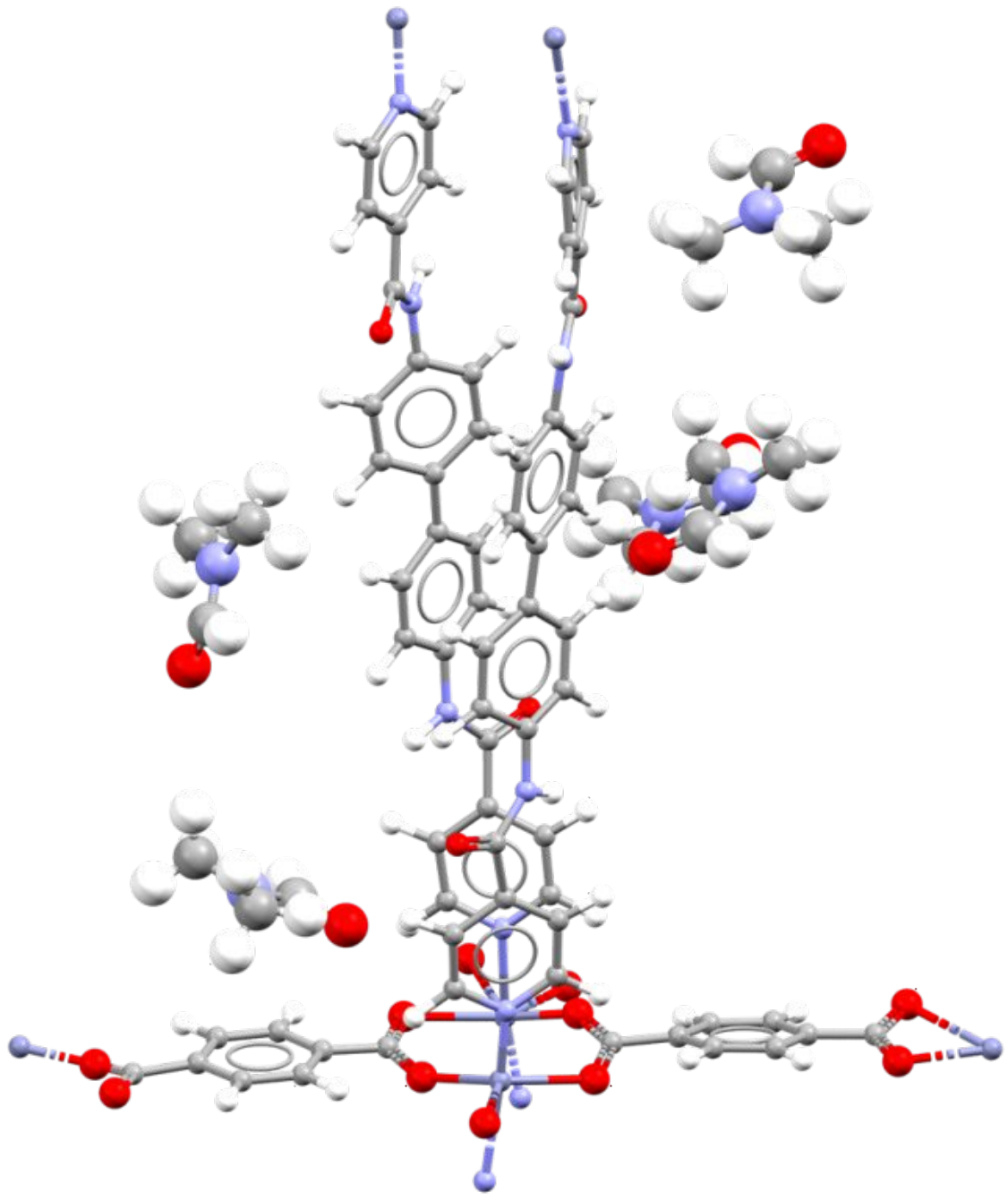

**Figure S1.** ORTEP visualization of the asymmetric unit of **PUM198-m**. All atoms are shown as isotropic ellipsoids at the 50% probability level and reported according to the color code C=grey, N=blue, O=red, Zn=light blue, H=white. Dotted bond represent the directions of polymeric expansion.

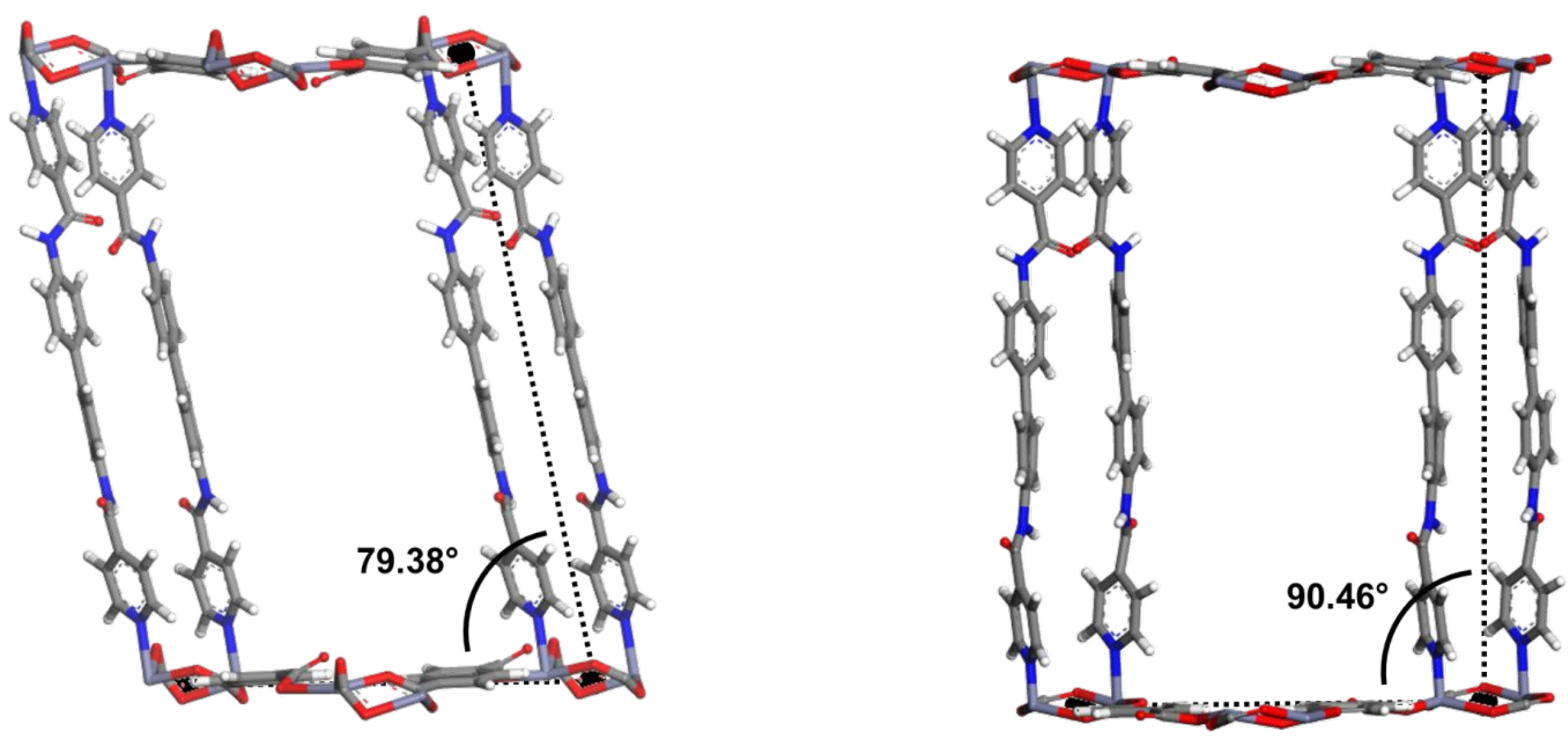


**Figure S2.** Comparison of a single framework net of **PUM198-m** as refined from 3DED (left) and from Rietveld refinement (right). The net is shown along the b axis (ac plane) in order to better display the tilting angle of the pillars, which are highlighted in dotted lines. The reported angles are constructed from centroids between Zn metals in the SBUs. Atoms are reported in stick style with the color code C=grey, N=blue, O=red, Zn=light blue, H=white.

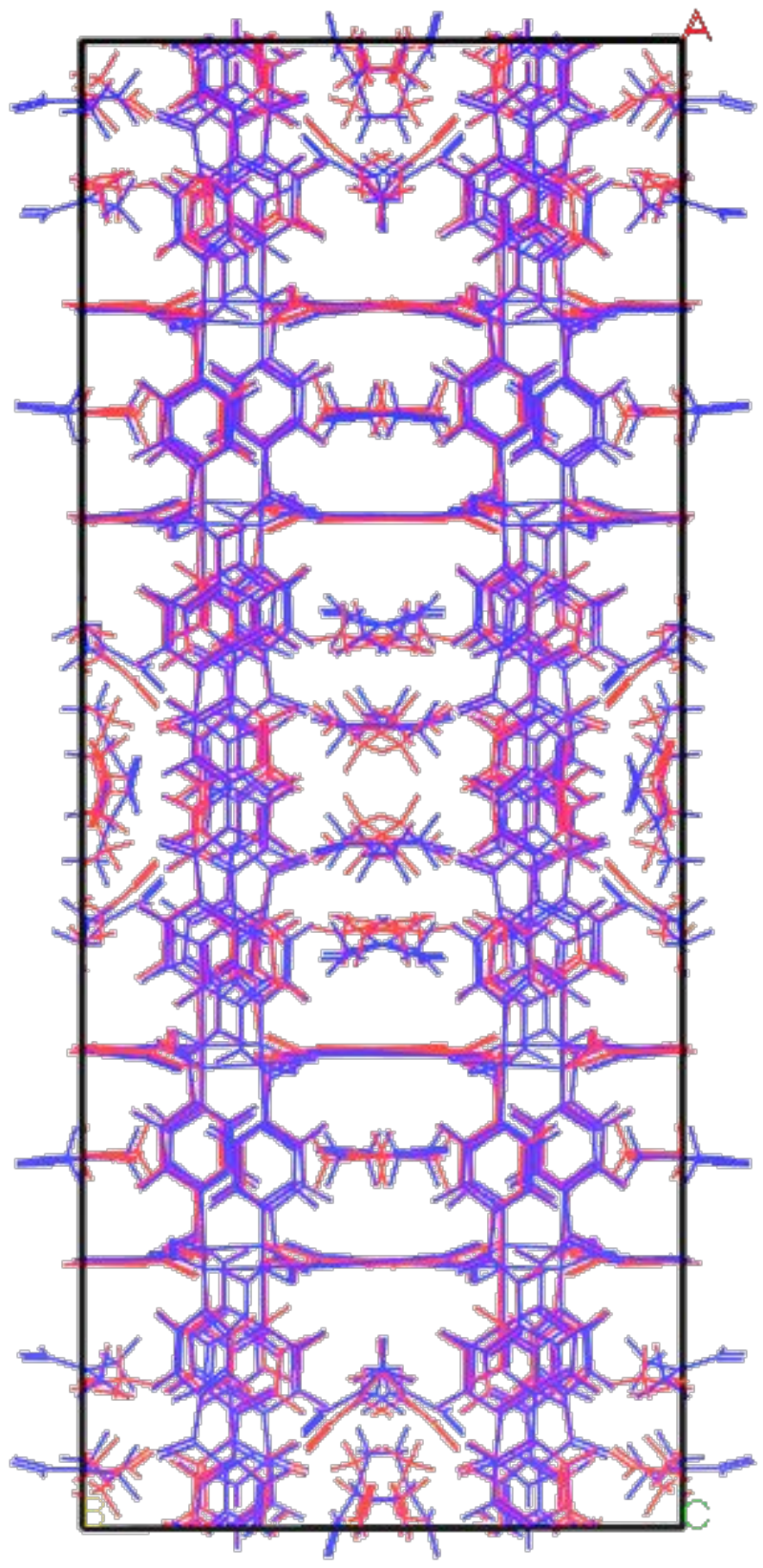


**Figure S3.** Comparison between structure of **PUM198-m** as optimized from PW-DFT (blue) and their Rietveld-refined counterpart (red).

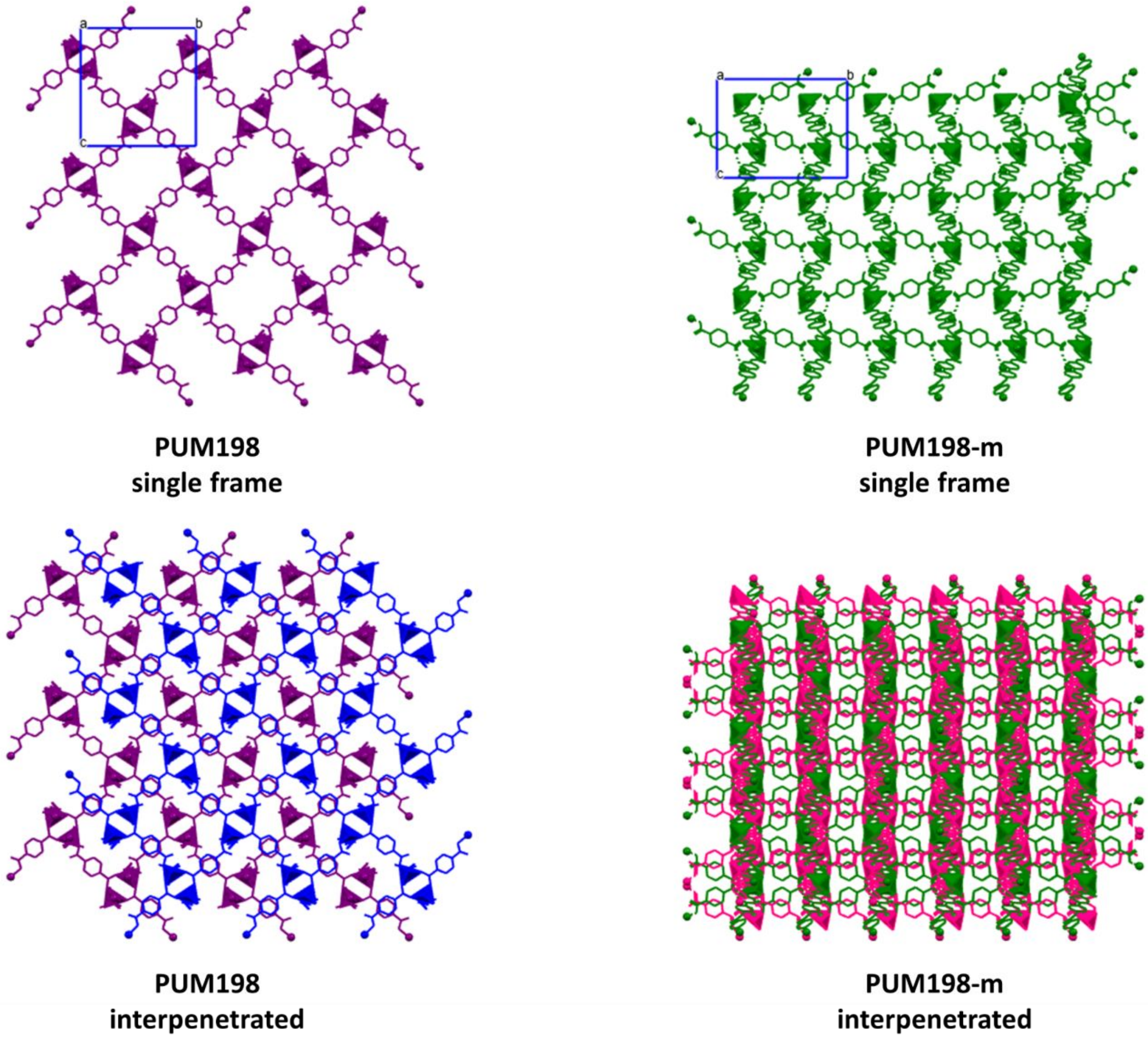


**Figure S4.** Comparison between **PUM198** and **PUM198-m** viewed along *a* crystallographic axis. The frameworks are shown both as a single network (top) and as a twofold interpenetrated architecture (bottom).

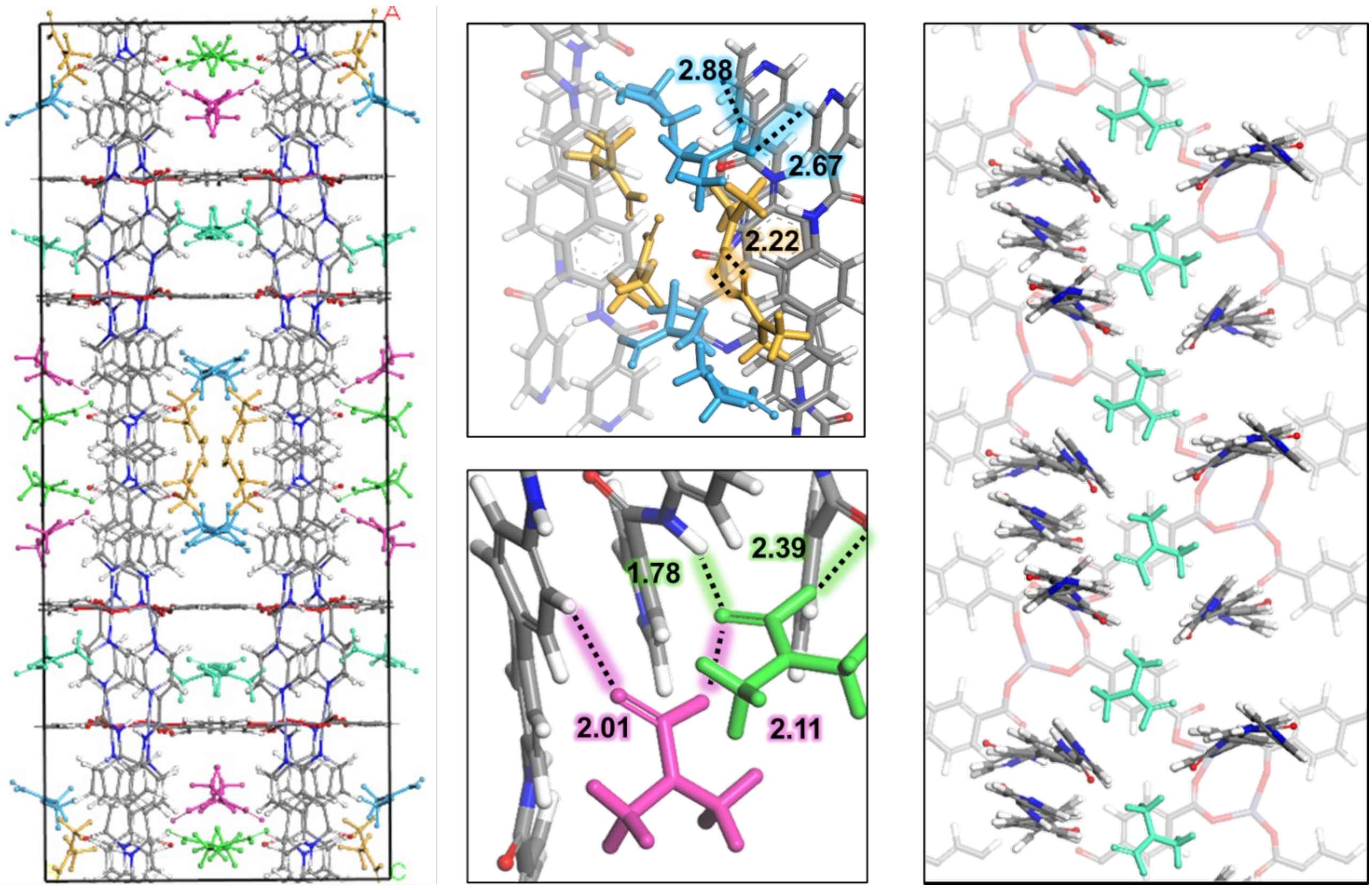


**Figure S5.** Details of the DMF molecules in the unit cell and details of their interactions with the framework. Symmetry-dependent DMF molecules are reported in the same color, and so are the dotted lines and the distances.

## Structural analysis of PUM198-m

The coordinative environment of the Zn atoms is the same trigonal bipyramid found in **PUM198**, albeit distorted: trigonal bipyramid involves three O atoms from three different dicarboxylates and two N atoms from two different bis-pyridinic ligands. Two carboxylate groups bridge two $Zn^{2+}$ centres to form a $[Zn_2(COO)_2]$ unit, while other two carboxylates bind each a Zn atom in a $k^1$-fashion. Within the *bc* plane terephtalates bind the metal atoms producing rhombic planar layers, with sides of 12.52 Å and angles of 76.91 and 103.09°. The coordination of the metal atom is completed by two apical pillars perpendicular to the carboxylate plane.

**PUM198-m** is two-fold interpenetrated. In this case, the pillar of the B net sits approximately in the centre of the mesh generated by the terephthalate ligands, interacting with them *via* N-H···O-C hydrogen bonds involving the amide groups of the pillar and the dangling carbonyl group of the terephthalate (the N···O and C···O distance are in all cases around 2.8 Å). Those hydrogen bonds give rise to two distinct rectangular channels running along the *c* direction (Figure 2c). The larger channels have a window of about 18.35 x 9.79 $Å^2$ while the smaller have a window of about 6.62 x 9.82 $Å^2$. The overall void volume of the structure is 32%, closely matching that of pristine **PUM198** (31%), indicating that the framework twisting does not significantly affect the total porosity. However, the different arrangement of the ligands modifies the functional groups exposed to the channels with respect to **PUM198** (Figure 1a and 2c). Each channel is fully occupied by five independent molecules of DMF. In the smaller channels, these are aligned parallel to the carboxylate layers, further strengthening the interaction between the two nets. In the bigger channels, by contrast, DMF molecules display two different distributions along direction *b*. In the narrowest sections of the channel, they are distributed parallel to the carboxylate planes, whereas in the widest sections they are tilted of about 45° with respect to the layers. In all cases, DMF is engaged in strong hydrogen bonds with the amidic moieties of the pillaring ligands.

## Polymorphs stability testing

**PUM198-m** stability was systematically evaluated under various conditions (Fig S11). Prolonged milling up to 4 h induced no detectable changes in crystallinity or phase composition. Likewise, storage in ambient atmosphere for six months produced no observable alteration. Thermal stability was further assessed by annealing the freshly synthetized powder at 120 °C for 8 hours under three different conditions: as air dried powder, in the sealed jar used for the mechanochemical synthesis without any additional treatment, and in the sealed jar after the addition of DMF (Figure S12). In all cases, no evidence of phase transformation to the solvothermal polymorph **PUM198** was observed. These results demonstrate that **PUM198-m** is thermodynamically stable under the tested conditions and does not convert to the alternative polymorph, even in the presence of DMF.

## Powder X-Ray Diffraction Analyses

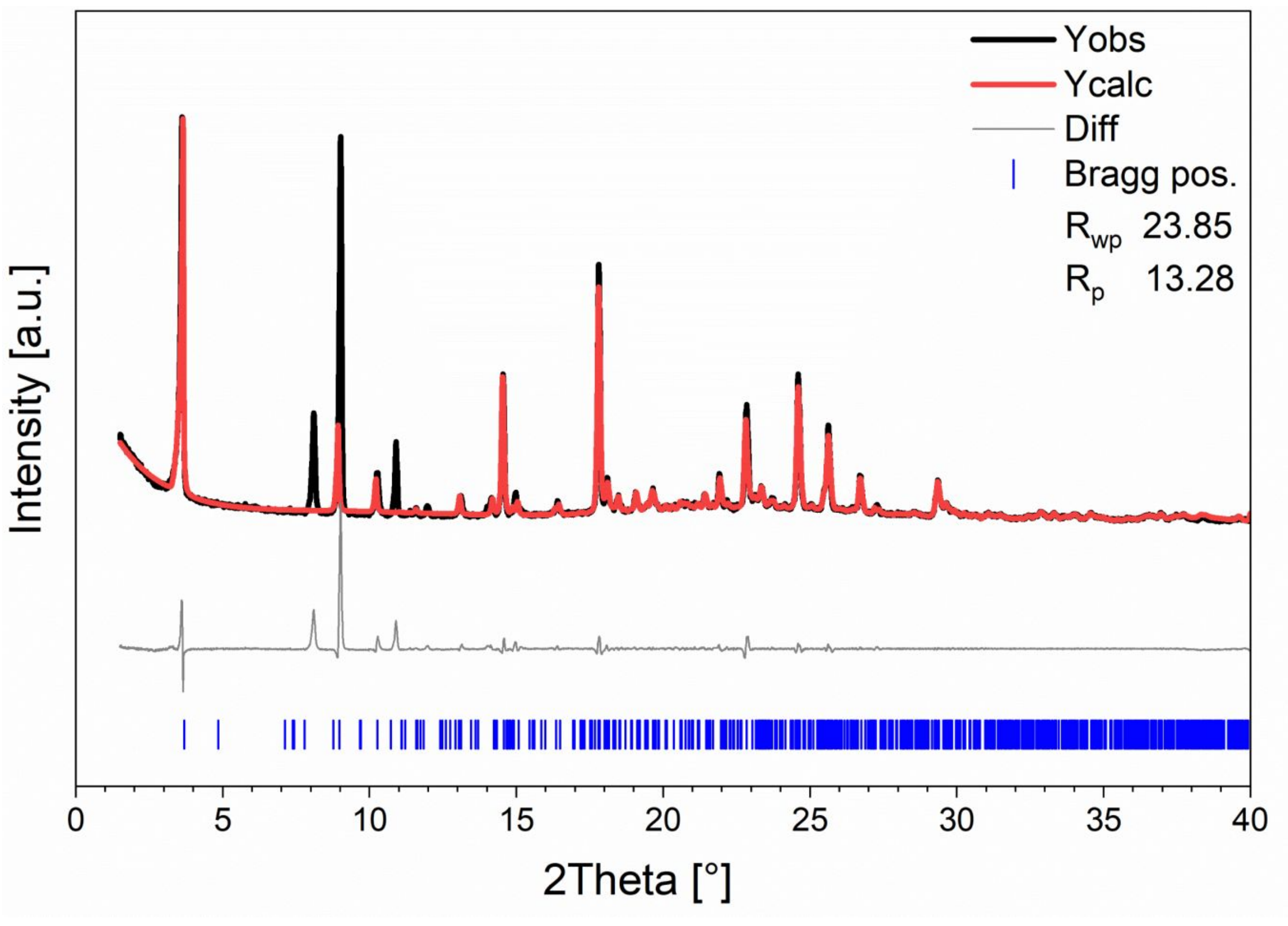


**Figure S6.** Pawley fit of experimental PXRD with the unit cell as derived from 3DED experiment. Experimental pattern ($Y_{obs}$, black line), simulated profile ($Y_{calc}$, red line) and $Y_{obs\text{-}calc}$ differential pattern (gray line). Blue ticks reported the *hkl* positions of the Bragg reflections. Rwp and Rp values are reported.

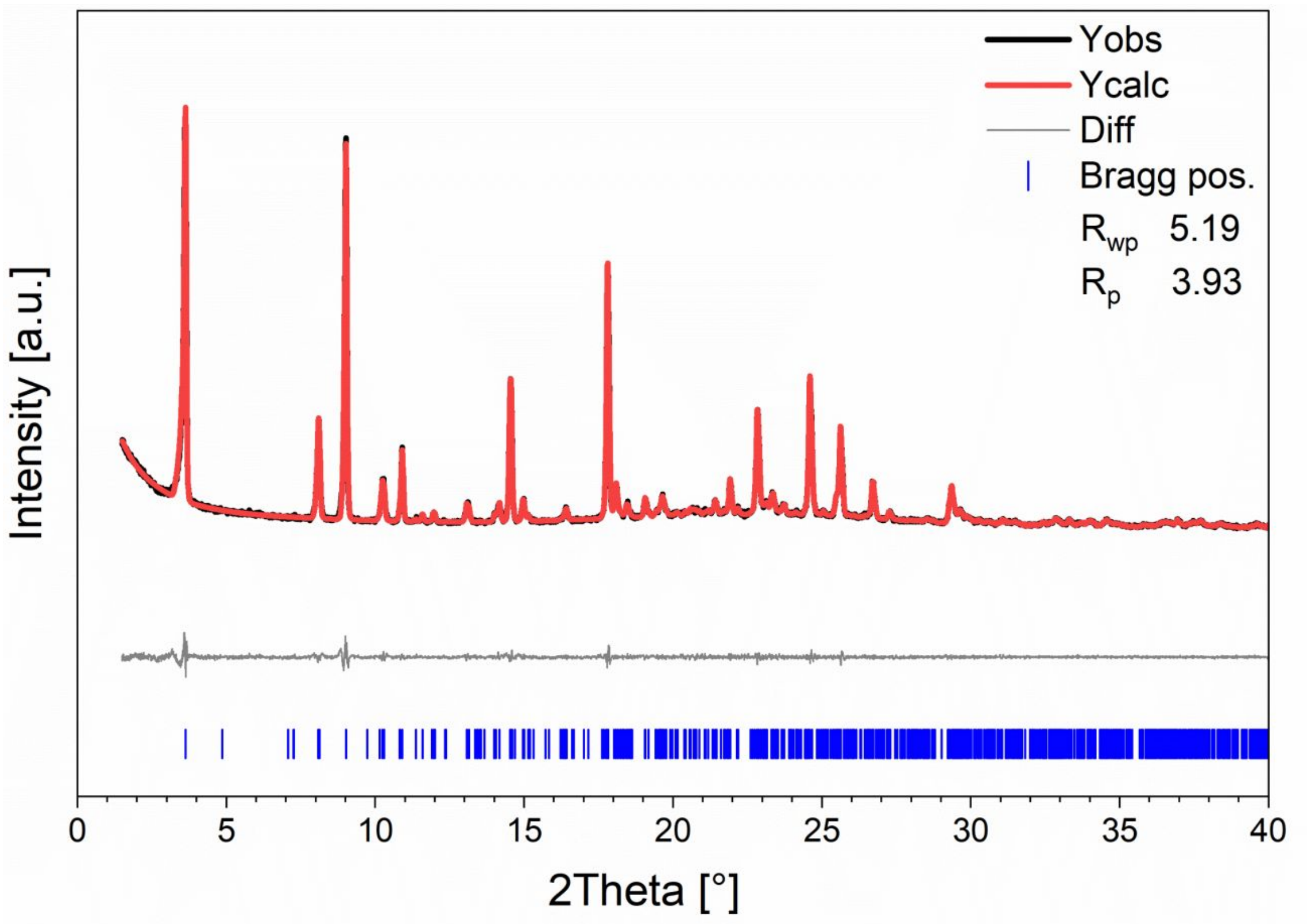


**Figure S7.** Pawley fit of experimental PXRD with the optimized unit cell parameters. Experimental pattern ($Y_{obs}$, black line), simulated profile ($Y_{calc}$, red line) and $Y_{obs-calc}$ differential pattern (gray line). Blue ticks reported the *hkl* positions of the Bragg reflections. Rwp and Rp values are reported.

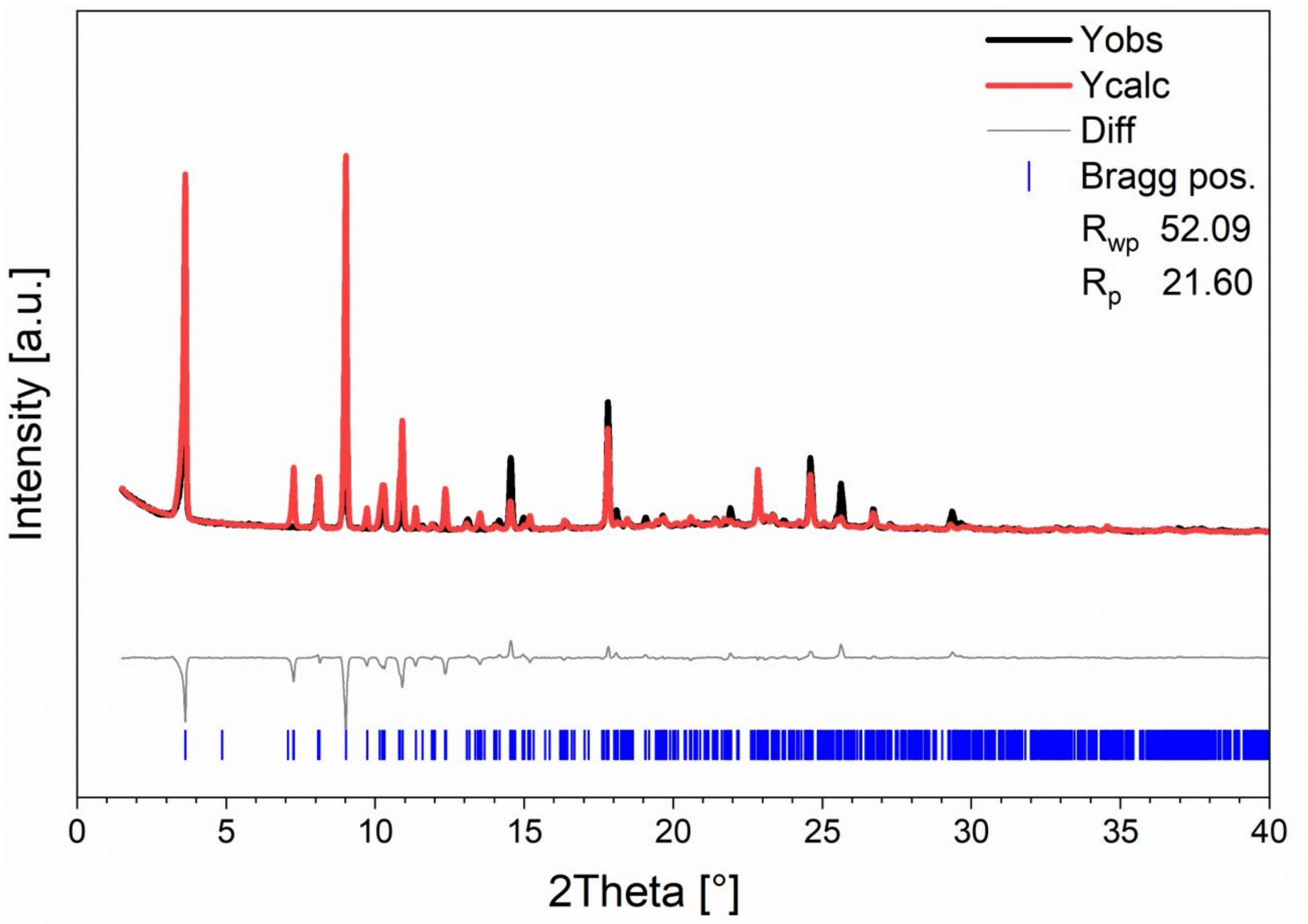


**Figure S8.** Rietveld fit of experimental PXRD with the DFT-optimized structure without the solvated DMF molecules. Experimental pattern ($Y_{obs}$, black line), simulated profile ($Y_{calc}$, red line) and $Y_{obs-calc}$ differential

pattern (gray line). Blue ticks reported the *hkl* positions of the Bragg reflections. Rwp and Rp values are reported.

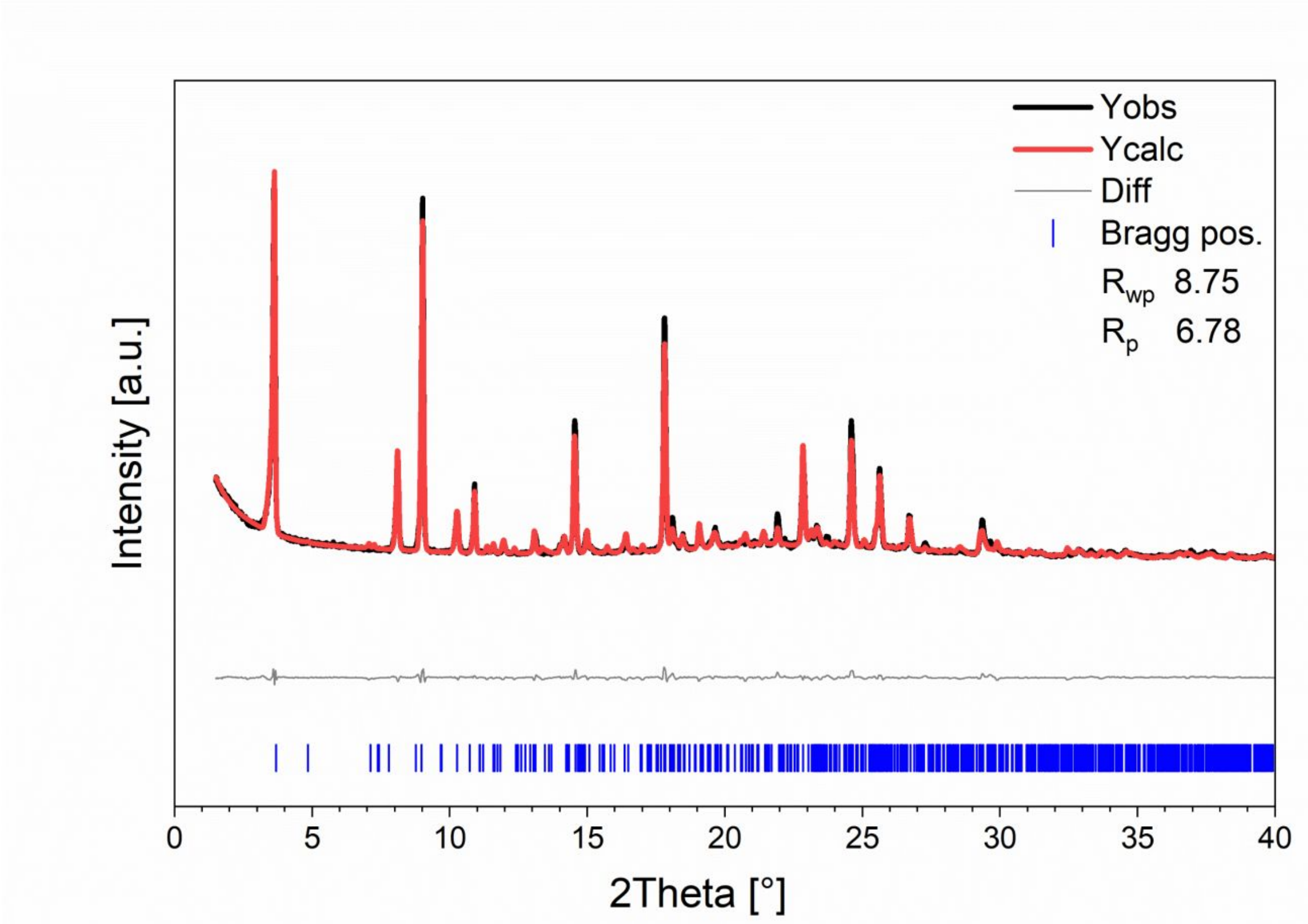


**Figure S9.** Rietveld fit of experimental PXRD with the DFT-optimized structure with the solvated DMF molecules. Experimental pattern ($Y_{obs}$, black line), simulated profile ($Y_{calc}$, red line) and $Y_{obs-calc}$ differential pattern (gray line). Blue ticks reported the *hkl* positions of the Bragg reflections. Rwp and Rp values are reported.

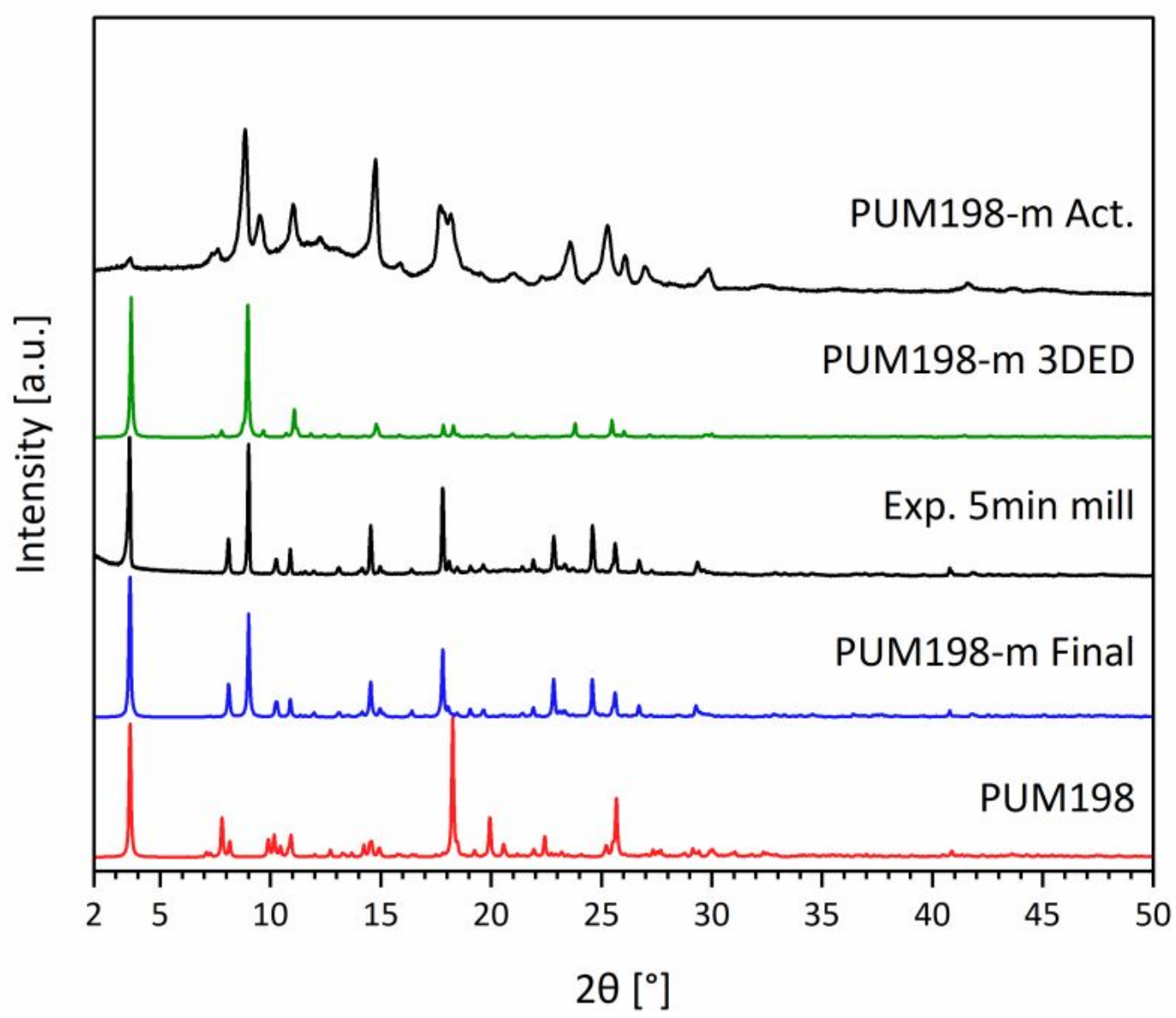


**Figure S10.** Comparison between experimental and simulated PXRD patterns. From bottom to top: **PUM198** simulated from the reported crystal structure (CCDC refcode: ZILDEG); **PUM198-m** simulated from the final DFT-optimized/Rietveld-refined ("Final") structure; the experimental PXRD pattern of **PUM198-m** after 5 min

of milling (capillary); **PUM198-m** simulated from the 3DED structure solution; **PUM198-m** after activation (12hrs under vacuum at 120°C, Bragg-Brentano).

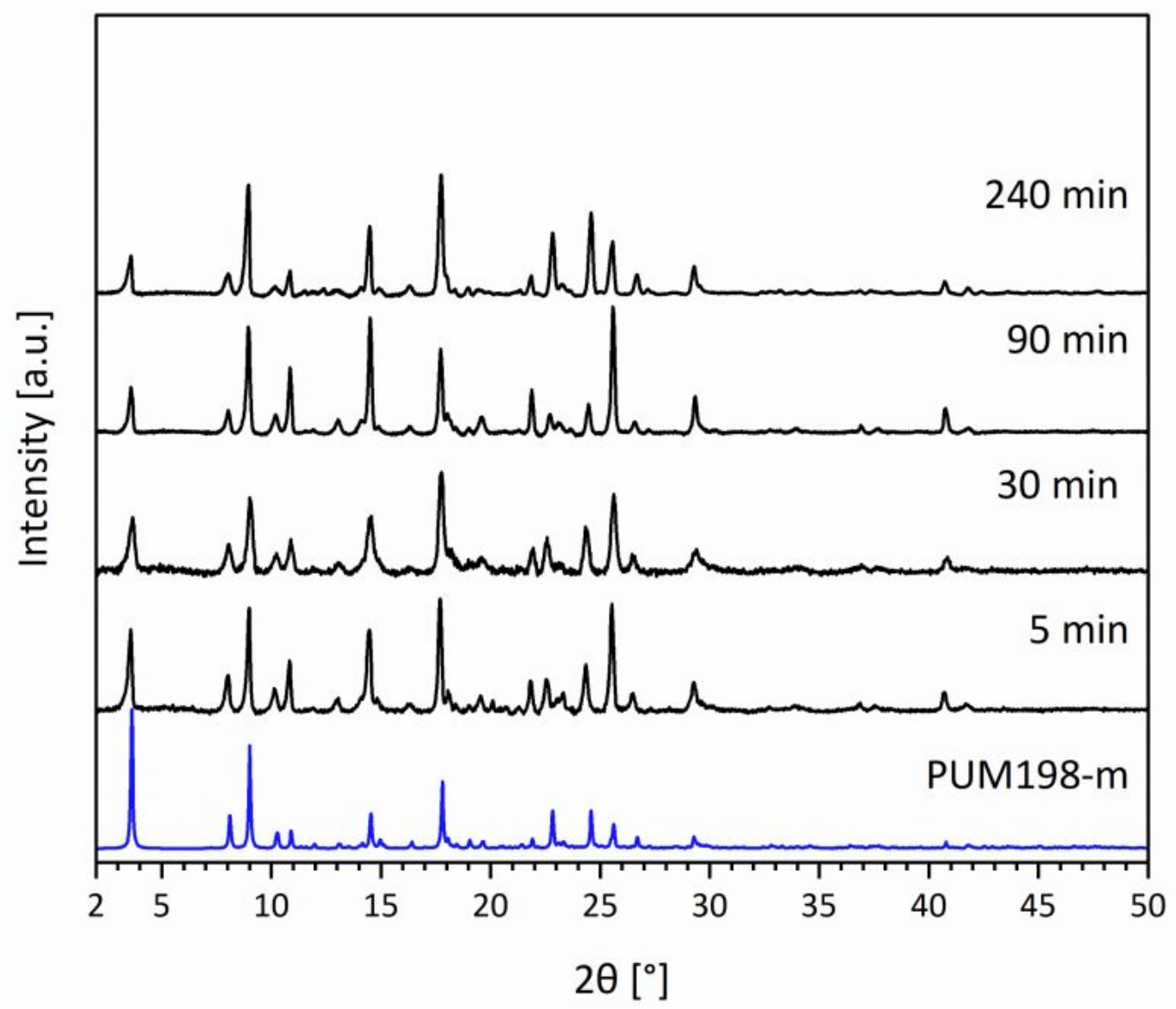


**Figure S11.** Mechanochemical synthesis of **PUM198-m** at different milling times and comparison with the calculated pattern from the final DMF containing structure of **PUM198-m** (blue). Note that the experimental PXRD were taken in a Bragg-Brentano Geometry, so relative intensities might be effected by sample preparation.

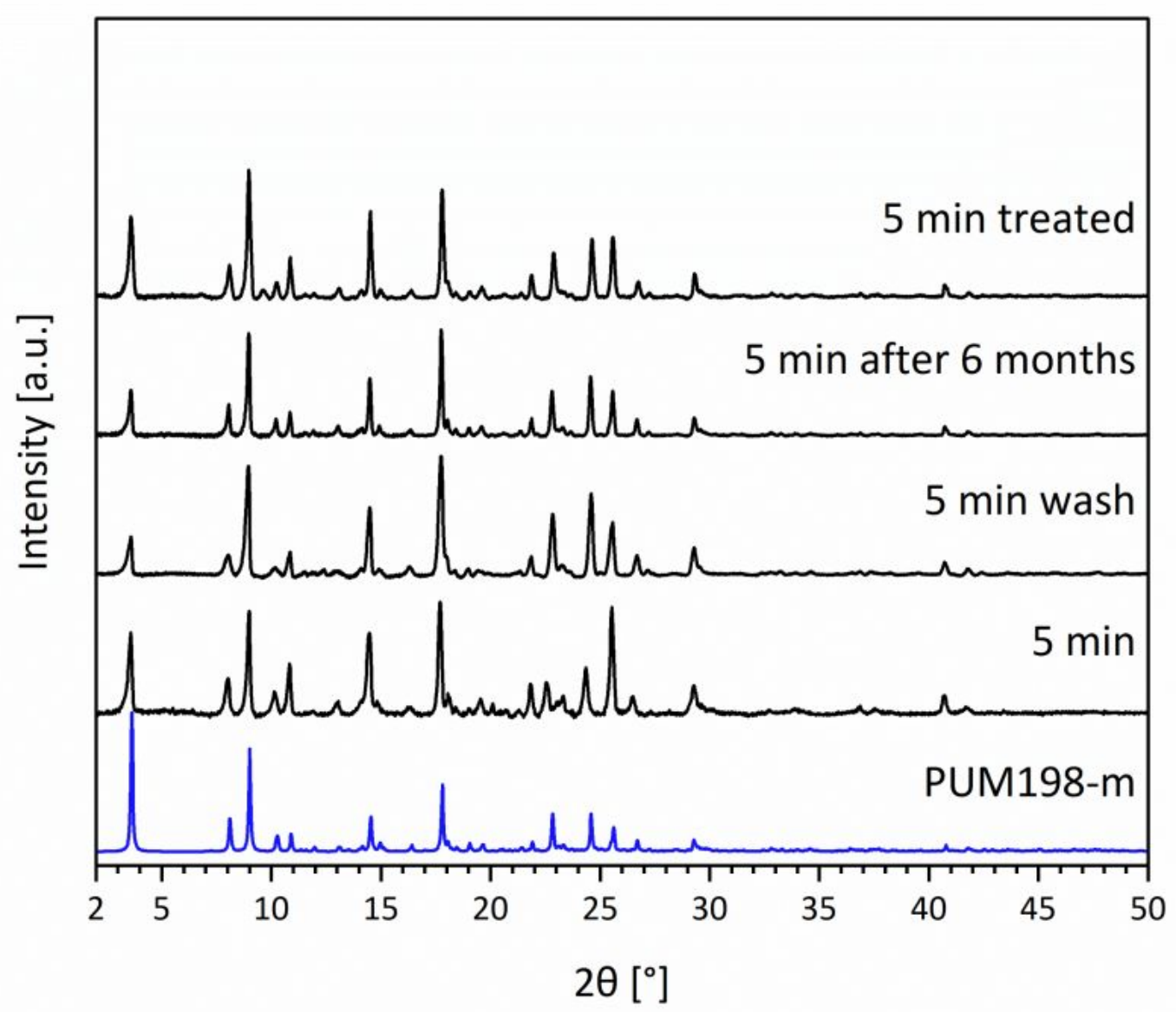


**Figure S12.** Comparison of **PUM198-m** synthesized via mechanochemistry: as-synthesized sample, after DMF washings, after 6 months of storage in a sealed vial, and after post-synthetic heat treatment in DMF. The experimental PXRD patterns are compared with the calculated pattern derived from the DMF-containing crystal structure of **PUM198-m** (blue). Note that the experimental PXRD were taken in a Bragg-Brentano Geometry, so relative intensities might be effected by sample preparation.

## $^1$H-NMR analysis

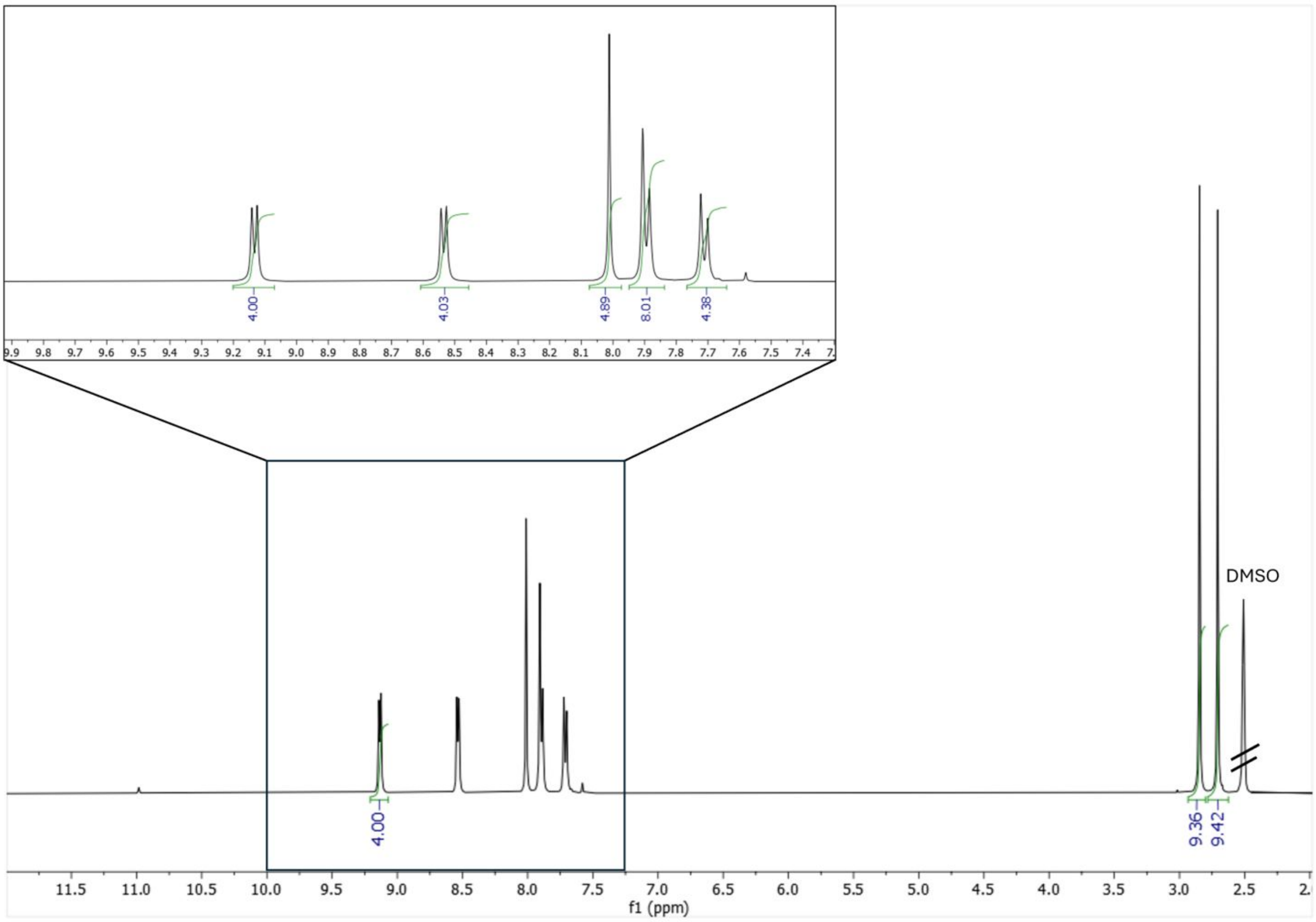


**Figure S13.** $^1$H-NMR analysis of **PUM198-m** was employed to determine solvent content. By comparing the integration of the ligand signals (normalized to one asymmetric unit) with those of the DMF resonances, a ratio of 4.89 (approx. 5) DMF molecules per asymmetric unit was estimated.

## Thermogravimetric analysis

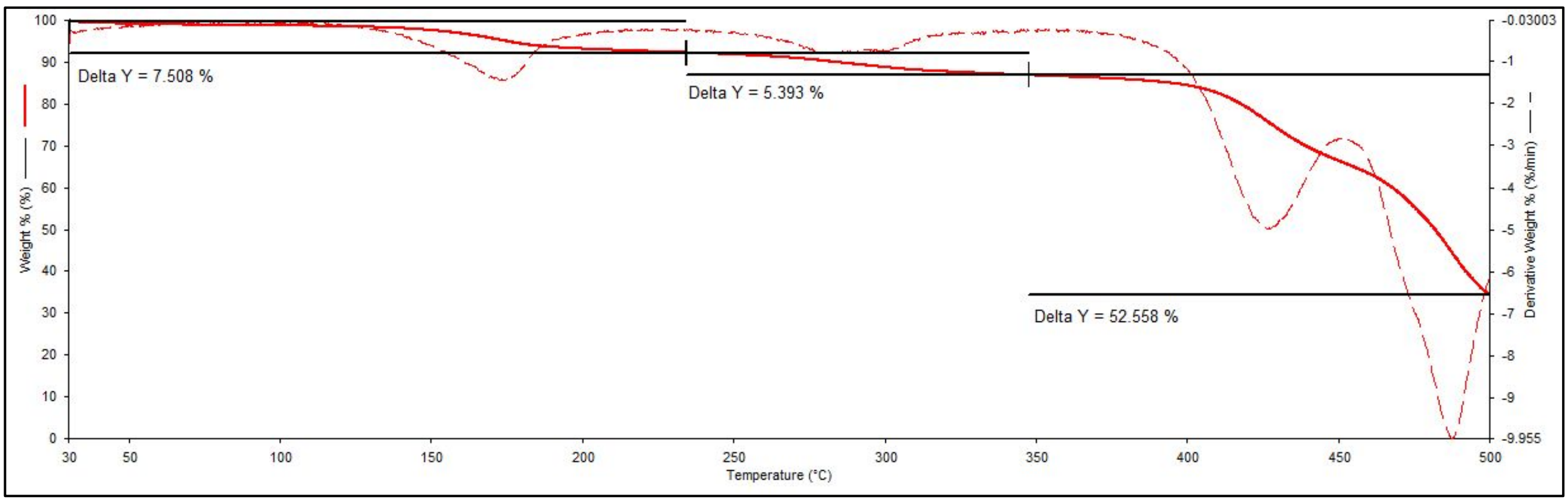


**Figure S14.** Thermogravimetric analysis (Air, 10 °C·min⁻¹) of **PUM198-m**. Two main solvent-loss steps are observed: 7.508% mass loss at ≈170 °C and 5.393% at ≈260 °C.; these steps correspond approximately to the loss of ~2.9 and ~2.1 DMF molecules, respectively. Framework decomposition occurs at about 400 °C.

## Topological details

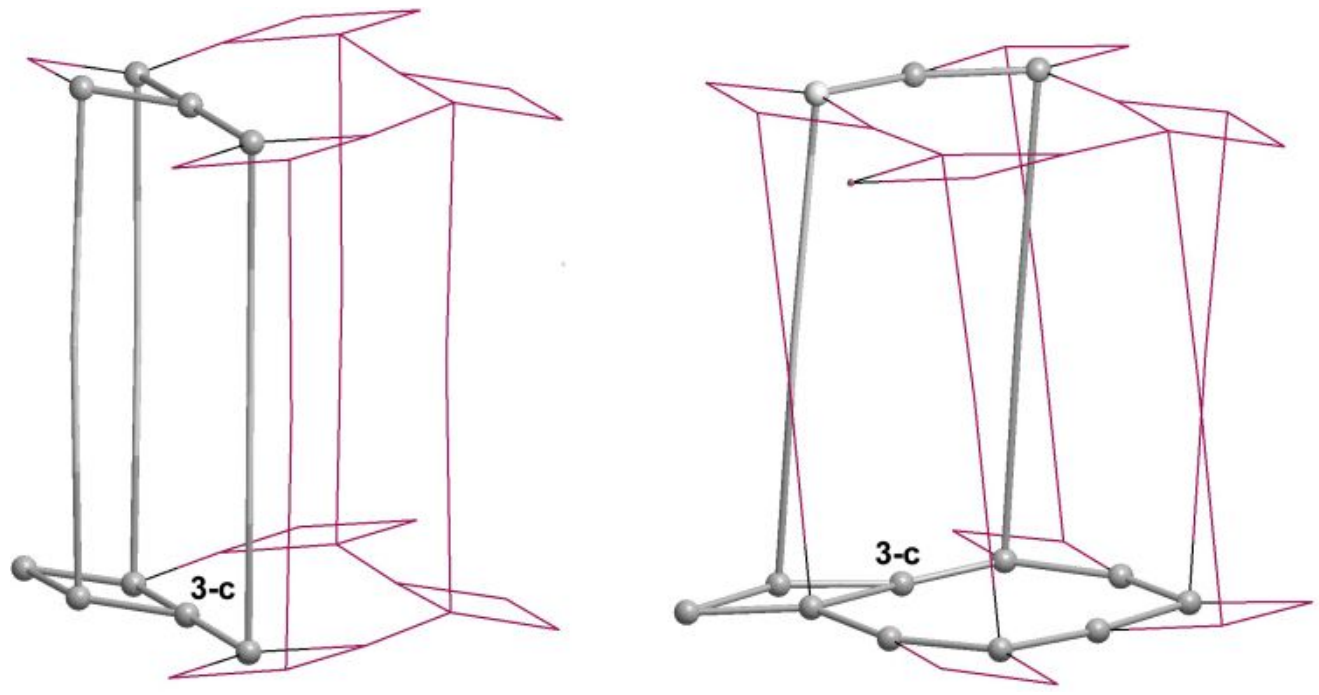


**Figure S15.** Details of the topological description of the 3-conn nodes of the **fet** net in **PUM198** (left) and of the **fsc**-3,5-Ibam net in **PUM198-m** (right).

## $N_2$ Adsorption Isotherms

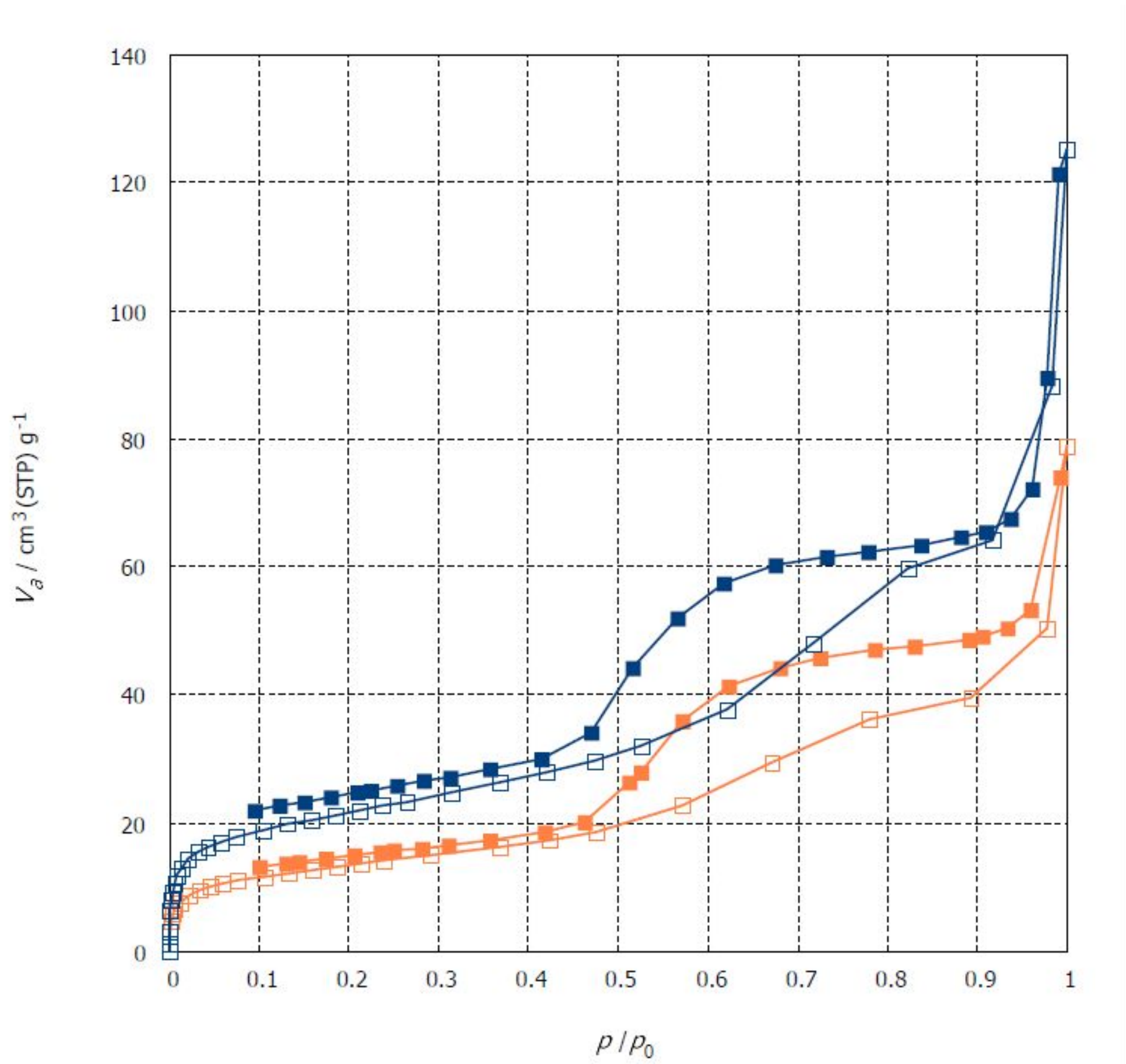


**Figure S16** $N_2$ adsorption–desorption isotherms measured at 77 K for **PUM198-m** (blue) and **PUM198** (orange). PUM198-m displays a slightly higher adsorbed volume over the entire relative pressure range. Open and filled symbols correspond to the adsorption and desorption branches, respectively. BET surface area was determined in the range $0.01 < p/p_0 < 0.1$.

## Structural comparison between consecutive resolution steps

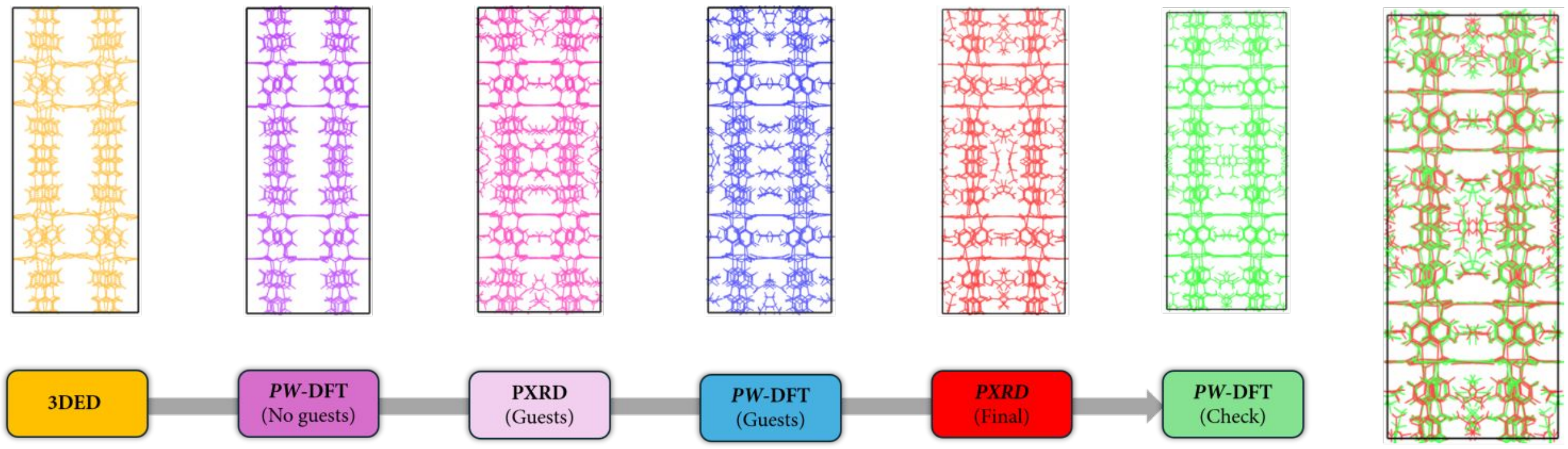


**Figure S17.** Comparison of the **PUM198-m** structure at different stages of structural resolution. From left to right: the structure obtained from 3DED (yellow); the structure after the first PW-DFT optimization cycle without DMF molecules included (purple); the structure after Rietveld refinement, using the previous model as the starting point and introducing DMF molecules whose positions were refined by the Rietveld method (pink); the structure after a subsequent PW-DFT optimization based on the DMF-containing model (blue); the structure after the final Rietveld refinement (red); and the structure after the final PW-DFT optimization (green). The far-right panel shows a direct comparison between the final Rietveld-refined structure and the final PW-DFT-optimized structure.

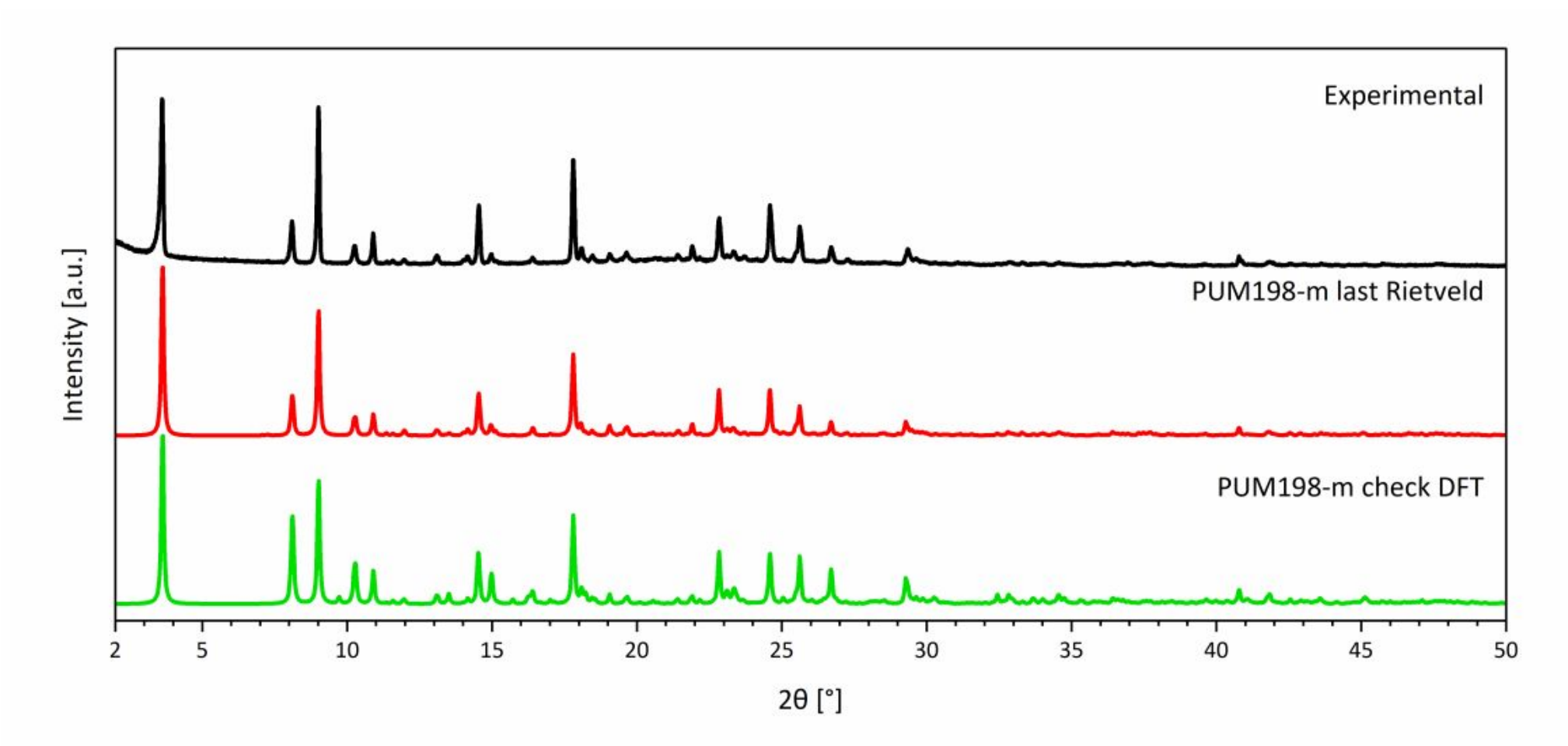


**Figure S18** Comparison of **PUM198-m** powder X-Ray diffraction calculated from the final PW-DFT optimization (green); the one calculated from the final Rietveld Refinement (red) and the experimental (black).

## References


[1] A. A. Coelho, "TOPAS and TOPAS-Academic: an optimization program integrating computer algebra and crystallographic objects written in C++" *J. Appl. Crystallogr.* **2018**, *51*, 210–218.

[2] M. Gemmi, E. Mugnaioli, T. E. Gorelik, U. Kolb, L. Palatinus, P. Boullay, S. Hovmöller, J. P. Abrahams, "3D Electron Diffraction: The Nanocrystallography Revolution" *ACS Cent. Sci.* **2019**, *5*, 1315–1329.

[3] A. Lanza, E. Margheritis, E. Mugnaioli, V. Cappello, G. Garau, M. Gemmi, "Nanobeam precession-assisted 3D electron diffraction reveals a new polymorph of hen egg-white lysozyme" *IUCrJ* **2019**, *6*, 178–188.

[4] E. Mugnaioli, T. Gorelik, U. Kolb, "'Ab initio' structure solution from electron diffraction data obtained by a combination of automated diffraction tomography and precession technique" *Ultramicroscopy* **2009**, *109*, 758–765.

[5] L. Palatinus, P. Brázda, M. Jelínek, J. Hrdá, G. Steciuk, M. Klementová, "Specifics of the data processing of precession electron diffraction tomography data and their implementation in the program *PETS2.0*" *Acta Crystallogr. Sect. B Struct. Sci. Cryst. Eng. Mater.* **2019**, *75*, 512–522.

[6] M. C. Burla, R. Caliandro, B. Carrozzini, G. L. Cascarano, C. Cuocci, C. Giacovazzo, M. Mallamo, A. Mazzone, G. Polidori, "Crystal structure determination and refinement *via SIR2014*" *J. Appl. Crystallogr.* **2015**, *48*, 306–309.

[7] Y.-B. Zhang, J. Su, H. Furukawa, Y. Yun, F. Gándara, A. Duong, X. Zou, O. M. Yaghi, "Single-Crystal Structure of a Covalent Organic Framework" *J. Am. Chem. Soc.* **2013**, *135*, 16336–16339.

[8] L. Romagnoli, A. D'Annibale, E. Blundo, A. Patra, A. Polimeni, D. Meggiolaro, I. Andrusenko, D. Marchetti, M. Gemmi, A. Latini, "4,4′-(Anthracene-9,10-diylbis(ethyne-2,1-diyl))bis(1-methyl-1-pyridinium) Lead Iodide C30H22N2Pb2I6: A Highly Luminescent, Chemically and Thermally Stable One-Dimensional Hybrid Iodoplumbate" *Chem. Mater.* **2023**, *35*, 1818–1826.

[9] P. P. Das, E. Mugnaioli, S. Nicolopoulos, C. Tossi, M. Gemmi, A. Galanis, G. Borodi, M. M. Pop, "Crystal Structures of Two Important Pharmaceuticals Solved by 3D Precession Electron Diffraction Tomography" *Org. Process Res. Dev.* **2018**, *22*, 1365–1372.

[10] I. Andrusenko, V. Hamilton, A. E. Lanza, C. L. Hall, E. Mugnaioli, J. Potticary, A. Buanz, S. Gaisford, A. M. Piras, Y. Zambito, S. R. Hall, M. Gemmi, "Structure determination, thermal stability and dissolution rate of δ-indomethacin" *Int. J. Pharm.* **2021**, *608*, 121067.

[11], "Dassault Systèmes. (2023). BIOVIA Materials Studio (Versione 2023). Dassault Systèmes BIOVIA." **n.d.**

[12] S. J. Clark, M. D. Segall, C. J. Pickard, P. J. Hasnip, M. I. J. Probert, K. Refson, M. C. Payne, "First principles methods using CASTEP" *Z. Für Krist. - Cryst. Mater.* **2005**, *220*, 567–570.

[13] C. Adamo, V. Barone, "Toward reliable density functional methods without adjustable parameters: The PBE0 model" *J. Chem. Phys.* **1999**, *110*, 6158–6170.

[14] A. Tkatchenko, M. Scheffler, "Accurate molecular van der Waals interactions from ground-state electron density and free-atom reference data" *Phys. Rev. Lett.* **2009**, *102*, 6–9.

[15] V.A. Blatov, A.P. Shevchenko, D.M. Proserpio "Applied Topological Analysis of Crystal Structures with the Program Package ToposPro" *Cryst. Growth Des*. **2014**, *14*, 3576-3586.

[16] A.P. Shevchenko, A.A. Shabalin, I.Yu. Karpukhin, V.A. Blatov, " Topological representations of crystal structures: generation, analysisand implementation in the TopCryst system" Science and Technology of Advanced Materials: Methods, **2022**, 2:1, 250-265.

[17] D. Balestri, A. Bacchi, P. Scilabra, P. Pelagatti, "Extension of the Pd-catalyzed C N bond forming reaction to the synthesis of large polydentate ligands containing N H functions" *Inorganica Chim. Acta* **2018**, *470*, 416–422.
